# Tuning the thermoelectrical properties of anthracene-based self-assembled monolayers†
†Electronic supplementary information (ESI) available: Synthesis of the molecules, device fabrication and characterization, charge transport in all the devices, charge transfer characterization and the theoretical demonstration of molecular orbitals as well as the calculated transmission coefficient of gold/molecule/gold systems for all molecules. See DOI: 10.1039/d0sc02193h


**DOI:** 10.1039/d0sc02193h

**Published:** 2020-06-22

**Authors:** Ali Ismael, Xintai Wang, Troy L. R. Bennett, Luke A. Wilkinson, Benjamin J. Robinson, Nicholas J. Long, Lesley F. Cohen, Colin J. Lambert

**Affiliations:** a Physics Department , Lancaster University , Lancaster , LA1 4YB , UK . Email: c.lambert@lancaster.ac.uk ; Email: b.j.robinson@lancaster.ac.uk; b Department of Physics , College of Education for Pure Science , Tikrit University , Tikrit , Iraq . Email: k.ismael@lancaster.ac.uk; c Department of Chemistry , Imperial College London , MSRH , White City , London , W12 0BZ , UK . Email: n.long@imperial.ac.uk; d The Blackett Laboratory , Imperial College London , South Kensington Campus , London , SW7 2AZ , UK . Email: l.cohen@imperial.ac.uk

## Abstract

It is known that the electrical conductance of single molecules can be controlled in a deterministic manner by chemically varying their anchor groups to external electrodes. Here, by employing synthetic methodologies to vary the terminal anchor groups around aromatic anthracene cores, and by forming self-assembled monolayers (SAMs) of the resulting molecules, we demonstrate that this method of control can be translated into cross-plane SAM-on-gold molecular films. The cross-plane conductance of SAMs formed from anthracene-based molecules with four different combinations of anchors are measured to differ by a factor of approximately 3 in agreement with theoretical predictions. We also demonstrate that the Seebeck coefficient of such films can be boosted by more than an order of magnitude by an appropriate choice of anchor groups and that both positive and negative Seebeck coefficients can be realised. This demonstration that the thermoelectric properties of SAMs are controlled by their anchor groups represents a critical step towards functional ultra-thin-film devices for future molecular-scale electronics.

## Abstract 2

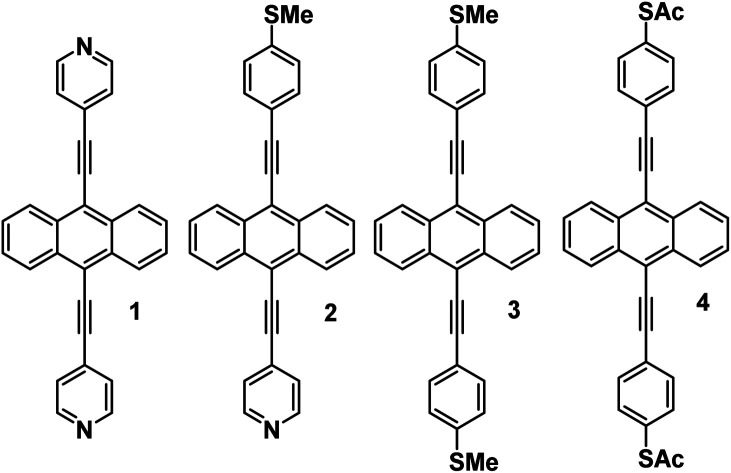
It is known that the electrical conductance of single molecules can be controlled in a deterministic manner by chemically varying their anchor groups to external electrodes.

Molecular electronic devices have the potential to deliver logic gates, sensors, memories and thermoelectric energy harvesters with ultra-low power requirements and sub-10 nm device footprints^1^–^4^ Single-molecule electronic junctions have been investigated intensively over the past few years, because their room-temperature electrical conductance is controlled by quantum interference (QI).^5^–^17^

As highlighted in recent reviews,^18^–^21^ Seebeck coefficients of single molecules can be controlled by varying the anchor groups,^22^–^24^ which bind them to external electrodes; Seebeck coefficients of single molecules with thiol anchor groups are found to be positive, while those with pyridyl anchor groups are measured to be negative, with room-temperature magnitudes, which are typically a few tens of μV K^–1^ at room temperature. Although a recent study^25^ shows nearly 2 orders of magnitude higher thermopowers than this value, the power generated by a single molecule is not yet sufficient to be of technological interest and therefore there is a need to demonstrate that this single-molecule tunability can be scaled up into thin films formed from self-assembled molecular arrays (SAMs).^26^

Here we demonstrate that in common with single molecules, the thermoelectric properties of SAMs can be tuned exquisitely by varying the choice of anchors groups, which bind the molecules to electrodes. Fig. 1 shows four molecules, in which electrical current is injected into and collected from an anthracene molecular core, *via* different anchor groups, formed from either pyridyl, thioether or thioacetate moieties. **1**, **3** and **4** are symmetric molecules, with the same anchor group at each end, while **2** is asymmetric and terminated by different anchors. In what follows, where appropriate, we refer to **1**, **2**, **3**, and **4** as 2Py, PySMe, 2SMe, and 2SAc, respectively.

**Fig. 1:**
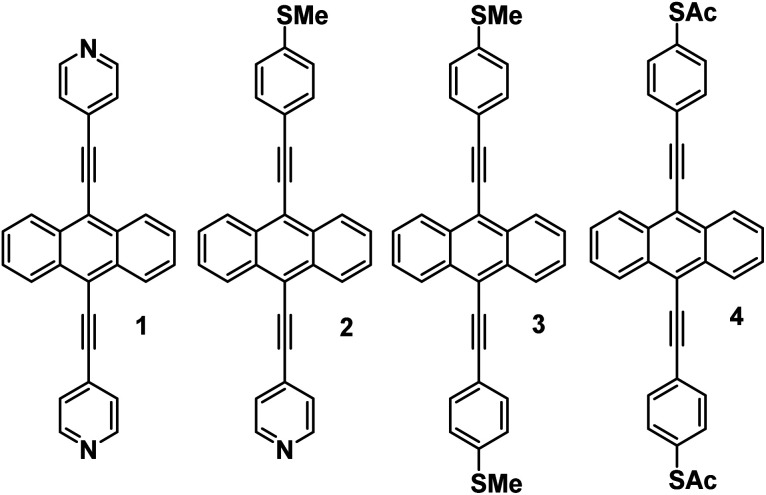
Chemical structures of molecular wires with anthracene cores. **1**, **2**, **3** and **4** correspond to the 7,2′ connectivity, **1** = 2Py, **2** = PySMe, **3** = 2SMe and **4** = 2SAc.

We have measured and calculated the electrical conductance of SAMs formed from **1–4** and have also measured and calculated their Seebeck coefficients. We shall demonstrate that both the sign and magnitude of the latter can be systematically improved by an appropriate choice of anchors.

## Synthesis

**Scheme 1:**
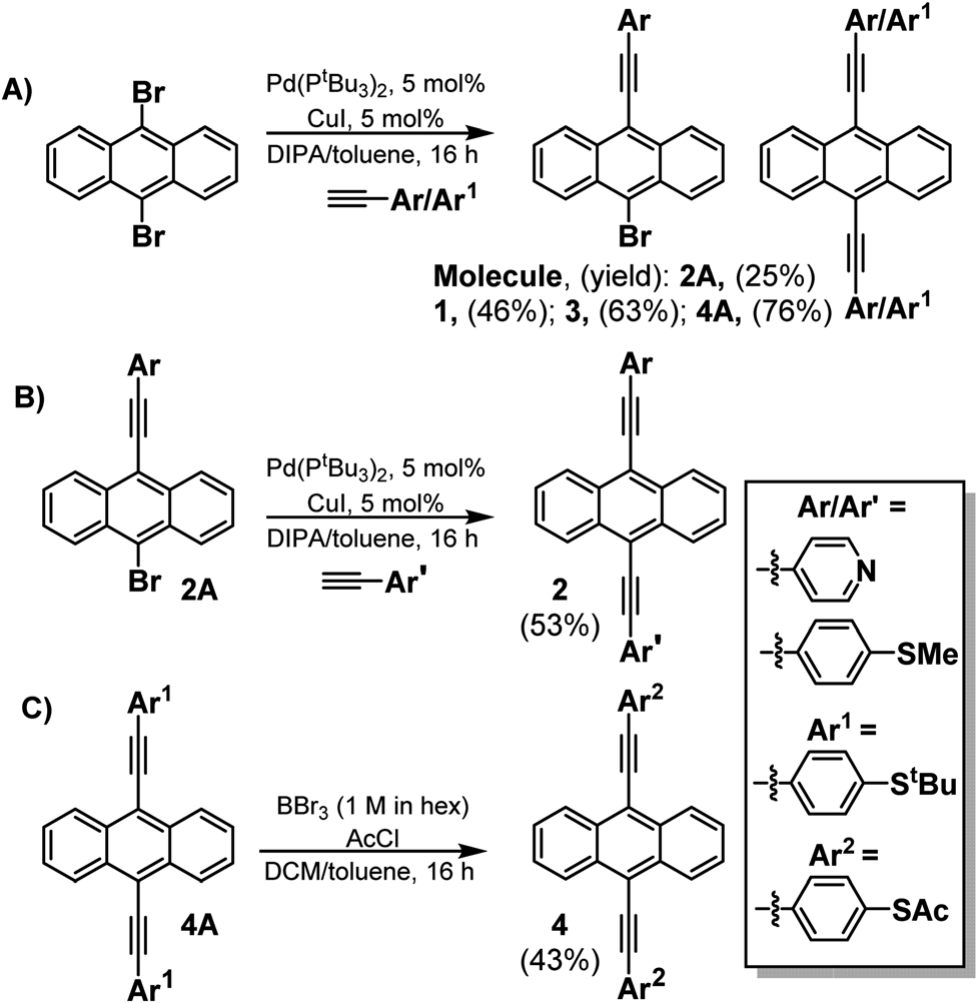
Synthesis of studied molecules. A representative synthetic pathway illustrating the construction of symmetrically disubstituted (A), asymmetrically disubstituted (B) and thioacetate-terminated (C) anthracenes.

This novel family of molecules (**1–4**) was synthesised by employing Sonogashira cross-coupling chemistry (Scheme 1) (For more details of their synthesis and characterization, see ESI Fig. S1–S12†). Initially, to form molecules **1–3**, dibromoanthracenes were reacted with the alkyne of choice (either 4-ethynylpyridine, or 4-ethynylthioanisole) to generate a mixture of the monosubstituted and symmetrically disubstituted products. While a mixture is always generated in the reaction, the ratio can be biased with control of reaction stoichiometry. The monosubstituted product can then be taken forward and coupled to the opposing alkyne to generate the asymmetrically disubstituted product (**2**). A different approach was taken for molecule **4** as 4-(ethynylphenyl)thioacetate undergoes a self-oligomerisation to form a cyclic trimer under Sonagashira conditions.^27^ To accommodate for this, a protecting-group strategy was adopted using the more stable 4-(ethynyl)phenyl-*tert*-butylthioether to form molecule **4A** (see page 6 of the ESI†), which could then be interconverted to molecule **4**. This was completed through the use of a boron tribromide dealkylation, followed by quenching with acetic anhydride to generate a terminal thioacetate. All compounds could be purified *via* flash column chromatography and were obtained in good yields (45–80%). Further details can be found in the ESI (Section 1.3†).

### SAM formation and characterization

Deposited molecular films were characterized by atomic force microscopy (AFM), which suggested the formation of high uniformity SAMs. All molecular films were grown on freshly prepared template stripped Au substrates^28^,^29^ with a surface roughness of 80–150 pm (see Methods section). Averaged roughness, as measured across multiple random areas (ESI-Table S1†), showed that SAMs of **2**, **3** and **4** conformationally follow the underlying gold surface with comparable roughness. However, SAMs of **1** show an increased roughness, which we attribute to the pyridyl-anchored molecules being able to form different adsorption geometries on Au substrates (Fig. 3 top panel).^30^ Film thicknesses were characterized by an AFM nano-scratching method^31^–^33^ (details explained in the ESI†) with the thickness of all the films in the range of 1.1–1.3 nm; this thickness corresponds to a monolayer of molecules in a perpendicular configuration, with a tilting angle of 30–60°. Larger area imaging of the sample surface further suggests that there are no multi-layered or un-covered regions; large scale uniformity was further confirmed through monitoring of film growth on a polished Au-coated quartz crystal microbalance (QCM). A Sauerbrey analysis of the QCM frequency change indicates, that in all cases, the molecular occupation area corresponds to that expected for a single molecule in a closely packed SAM^34^,^35^ (Table S1†).

**Fig. 2:**
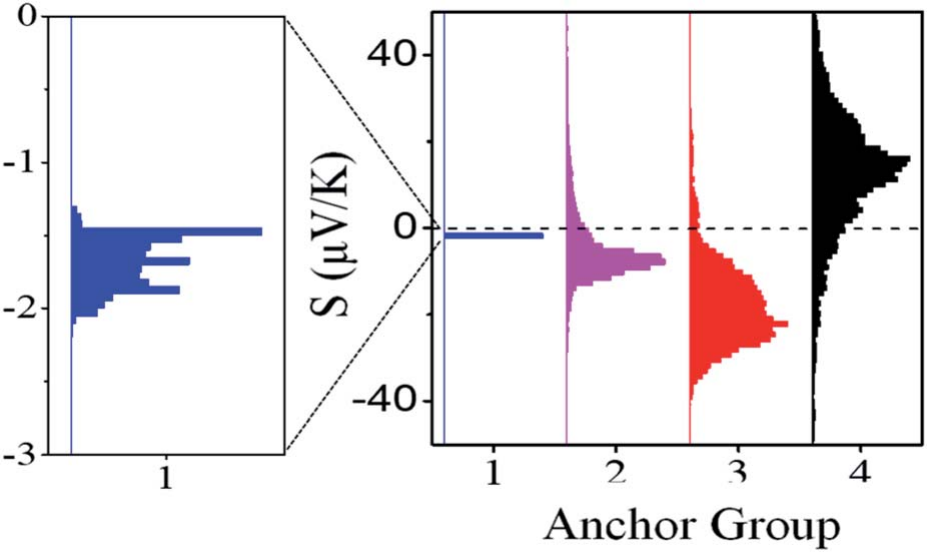
Experimental thermoelectrical properties of SAMs, the histogram of Seebeck coefficient distribution of **1**, **2**, **3** and **4**.

**Fig. 3:**
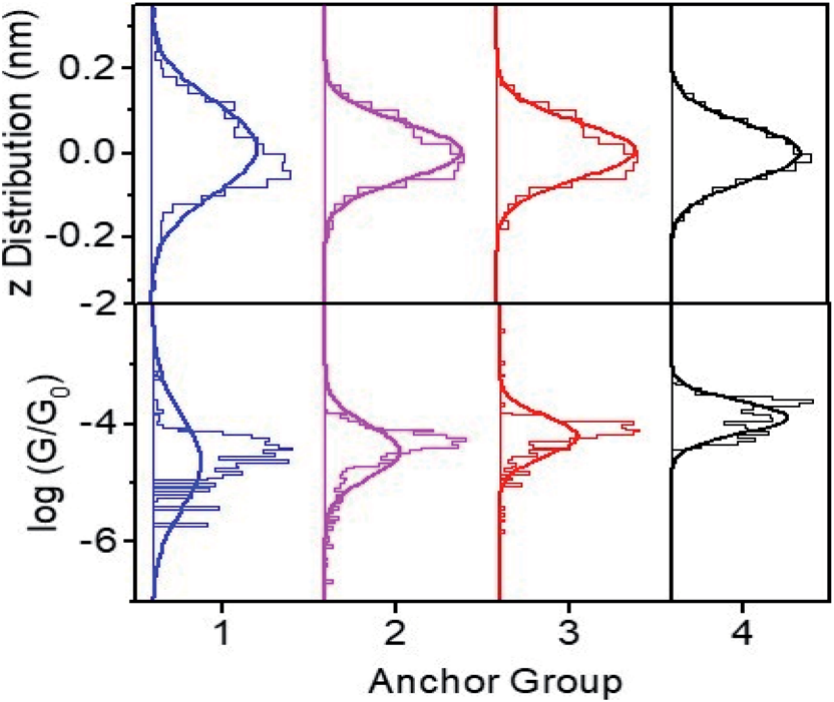
Plot of height distributions (upper panels) and log
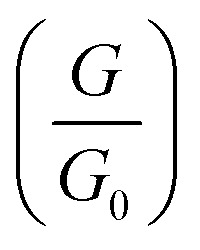
 distributions (lower panels) for molecules **1–4**. Each distribution is formed from at least 2000 measured points.

### Electrical and thermal characterization

Molecular conductance was characterized by conductive AFM (cAFM), where the number of molecules under the probe is estimated from the contact area between probe, sample surface (obtained *via* Hertz Model^36^–^38^) and the single-molecule occupation area obtained from QCM and AFM (Table 1).

**Table 1:** Experimental measurements and theoretical calculations (For **1**, **2** and **3**, *E*_F_ – *E*DFTF = –0.5 eV; for **4**, *E*_F_ – *E*DFTF = 0.5 eV. Theoretical values are obtained from the yellow plots in Fig. S29 and S30 of the ESI)^*a*^

M	Exp. (*G*/*G*_0_)	Std	Theo. (*G*/*G*_0_)	Exp. S (μV K^–1^)	Std	Theo. S (μV K^–1^)
**1**	3.24 × 10^–5^	5 × 10^–6^	2.1 × 10^–4^	–2.50	0.4	–5.5
**2**	4.54 × 10^–5^	3 × 10^–6^	3.5 × 10^–4^	–4.70	2.5	–12.0
**3**	7.01 × 10^–5^	9 × 10^–6^	1.66 × 10^–4^	–21.6	7.0	–20.0
**4**	1.28 × 10^–4^	5 × 10^–6^	1.59 × 10^–4^	+11.0	9.1	+12.5

^*a*^Further details about the thermoelectric measurements and the distribution of conductances of different SAMs are listed in the ESI (Experimental section).

Fig. S13 and S14† show plots of thermo-voltage *versus* temperature difference for the different SAMs. Fig. 2 shows a comparison between the resulting distributions of measured Seebeck coefficients of the different SAMs and reveals a systematic increase of the Seebeck coefficient across the series of molecules **1**, **2** and **3**. Furthermore, the introduction of thioacetate anchors in 2SAc (**4**) causes the Seebeck coefficient to change sign and become positive, whereas the Seebeck coefficients of the other three SAMs are negative.

Conductance distribution maps for molecules **1–4** are shown in Fig. S15,† and the resulting conductance distributions are shown in Fig. 3 (lower panels). These show that the systematic increase in the Seebeck coefficients of **1**, **2**, **3** is accompanied by a corresponding increase in their electrical conductances, with 2SAc (**4**) possessing the highest conductance.

The upper panels of Fig. 3 show the height distributions across the films and reveals that **1** has a broader height distribution and therefore higher degree of roughness than the others.

### Density functional theory

To compute the transport properties of molecules **1–4**, we used density functional theory combined with the quantum transport code Gollum^39^ to obtain the transmission coefficient *T*(*E*) describing electrons of energy *E* passing from the source to the drain electrodes *via* the anthracene cores (electronic structures and relaxed geometries of all molecules are shown in Fig. S18–S28† of the ESI). From *T*(*E*), the room temperature electrical conductance *G* and Seebeck coefficient *S* are determined, as shown in Fig. S29 and S30,† and as described in the Theoretical Methods section. By comparing *T*(*E*) for single molecules against that of a parallel array of several molecules, it was demonstrated recently^17^ that the *T*(*E*)for a SAM is approximately the same as that of a single molecule. Therefore in what follows, calculations are performed on single-molecule junctions.

Previous comparison between experiment and theory revealed that electron transport through polyaromatic hydrocarbons takes place near the middle of the energy gap between the highest occupied molecular orbital (HOMO) and the lowest unoccupied molecular orbital (LUMO),^40^ and indeed we find that the closest agreement between theory and experiment is obtained for a Fermi energy near the mid-gap, as indicated by the vertical dashed lines in Fig. S29.†

As expected, from literature studies of single molecules, **4** is HOMO dominated (due to the presence of thioacetate anchors), whereas **1**, **2** and **3** are LUMO dominated (due to the presence of thioether^26^ or pyridyl anchors). As demonstrated in Fig. S29 and S30,† since the sign of the Seebeck coefficient is determined by the slope of the transmission coefficient near the Fermi energy, this switching from HOMO to LUMO-dominated transport causes the sign of Seebeck coefficient to change. LUMO-dominate transport was also predicted for the thioether-terminated molecules in ref. 41, and measured experimentally for the longest molecule in ref. 42 and for the thioether-terminated anthracene in ref. 26.

**Fig. 4:**
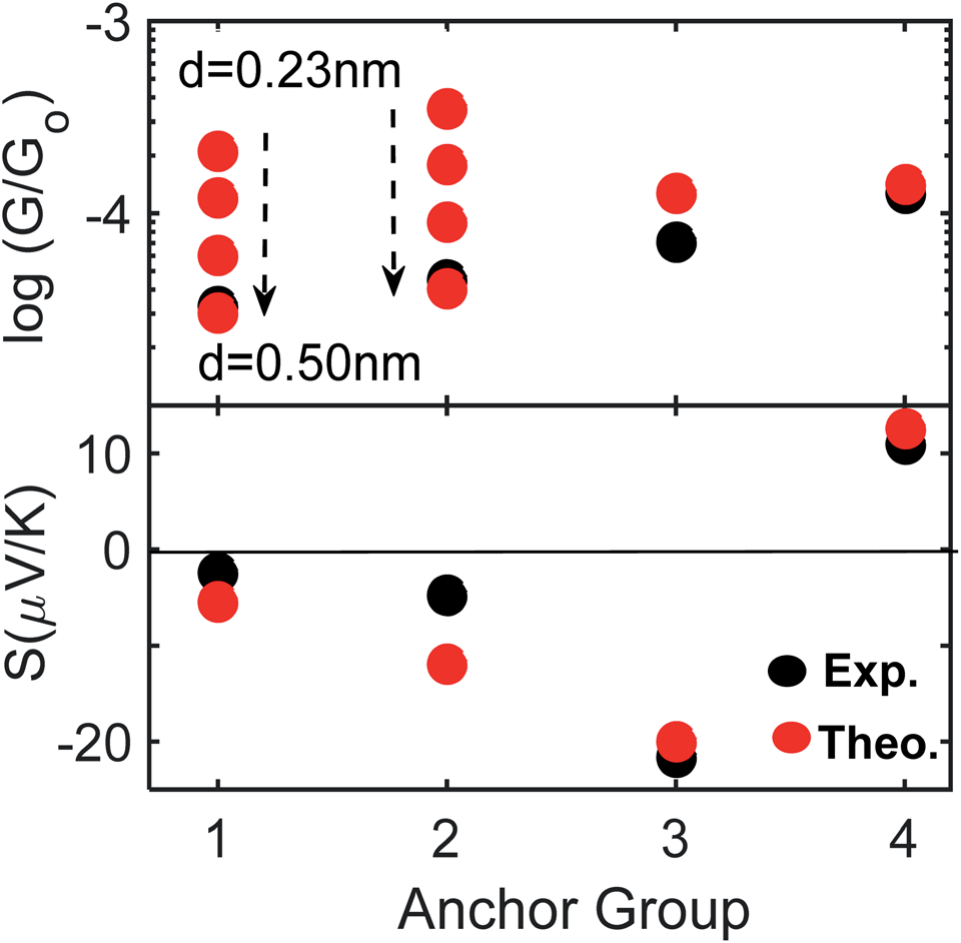
Electrical and thermoelectrical properties of **1–4**. A comparison between experiment and theory. For **1** and **2** the optimum distance is 0.23 and increases to 0.50 nm (black-arrow, also see Fig. 3 top panel and Fig. S32†).

Fig. 4 shows a comparison between experiment and theory for conductances and Seebeck coefficients. For **3** and **4** experimental and theoretical values for the conductance are in broad agreement. However, for SAMs based on **1** (2Py) and **2** (PySMe), the theoretical conductances computed using the theoretical optimum distance (of 0.23 nm) between the anchor groups and electrodes are significantly higher than the measured values. This occurs, because as shown in Fig. S16 and S17,† the film quality of these SAMs is poorer than that of the 2SAc-, 2SMe-based SAMs. Consequently, the actual anchor-electrode distances in these Py-terminated SAMs is greater than the optimum value and measured to be of the order of 0.50 nm. For 2Py and PySMe – terminated molecules, the top panel of Fig. 4 shows that increasing the anchor-electrode distance from 0.23 nm to 0.50 nm in a series of steps, successively decreases their electrical conductance and that at a distance of 0.50 nm, the computed conductances are close to the measured values. On the other hand, the lower panel shows that this has only a slight effect on their Seebeck coefficients (see Fig. S32†) and that excellent agreement between theory and experiment is retained (see the height distribution of different SAMs shown in Fig. 3 and the FWHM shown in Fig. S17†). As well as studying the dependence of the Seebeck coefficient (*S*) on anchor-electrode distances, we also varied the tilt angles of molecules (lower panel of Fig. S31 and S32†) and found the tilt angle has only a small effect on the computed Seebeck coefficients.

In summary, through the rational design, synthesis and implementation of a new family of molecules, we have demonstrated that the thermoelectrical performance of anthracene-based molecular films can be systematically tuned by varying the anchor groups connecting the molecules to electrodes. In particular the Seebeck coefficient of an anthracene-based SAM with SMe anchors is found to be an order of magnitude higher than that of a SAM with pyridyl anchors. This demonstrates that methods of controlling thermoelectric properties of single molecules^31^–^33^ can be transferred into SAMs and that significant boosts in thermoelectric performance can be achieved through a judicious choice of anchor groups. This method of controlling the thermoelectric properties of molecular films opens the way to new design strategies for functional ultra-thin-film materials and electronic building blocks for future integrated circuits. In particular, it means that strategies for optimising single-molecule transport properties using room-temperature quantum interference^5^–^16^ can be utilised in SAMs, both by engineering QI within the core structure,^17^ by varying the anchor group as shown here, and possibly by electrochemical means for achieving active control.

For the future, it would be of interest to demonstrate that methods for suppressing phonon transport in single molecules^43^ could also be transferred into SAMs, so that thermoelectric efficiency can be optimised by reducing their thermal conductance.

## Methods

### Compound synthesis and characterization

All reactions were performed with the use of standard air-sensitive chemistry and Schlenk line techniques, under an atmosphere of nitrogen. No special precautions were taken to exclude air during any work-ups. All commercially available reagents were used as received from suppliers, without further purification. 4-Ethynylthioanisole and 4-(ethynyl)phenyl-*tert*-butylthioether were synthesised through adapted literature procedures^44^,^45^ Solvents used in reactions were collected from solvent towers sparged with nitrogen and dried with 3 Å molecular sieves, apart from DIPA, which was distilled onto activated 3 Å molecular sieves.

^1^H and ^13^C1 NMR spectra were recorded on a Bruker Avance 400 MHz spectrometer and referenced to the residual solvent peaks of either CDCl_3_ at 7.26 and 77.2 ppm, respectively or DC_2_Cl_2_ at 5.32 and 53.8. Coupling constants are measured in Hz. Mass spectrometry analyses were conducted by Dr Lisa Haigh of the Mass Spectrometry Service, Imperial College London. Infrared spectra were recorded on a PerkinElmer Spectrum FT-IR spectrometer.

### SAMs fabrication

For QCM: The QCM substrate (International Crystal Manufacturing, USA) was rinsed by acetone (>99%), methanol (>99%) and iso-propanol (>99%) in series and cleaned by oxygen plasma for 5 minutes. The stabilised, initial resonance frequency (*f*_0_) of the cleaned QCM substrate was recorded. The cleaned QCM substrate was then immersed in 1 mM solution of molecules **1–4** in 1 : 2 ethanol : THF mixture (>99.9%, bubbling with nitrogen for 20 min to remove oxygen) from 12 hours to 48 hours. Optimised assembly times were established over multiple depositions. The substrate was subsequently rinsed by THF and ethanol several times to remove excess physisorbed molecules before drying in vacuum (10^–2^ mbar, 40 °C). The frequency of substrate after SAMs growth was again measured by the QCM. The equivalent measurement, where the QCM substrate was immersed in 1 : 2 ethanol : THF mixture without any molecules **1–4** present was also pre-formed as a reference.

### For SPM: TS gold preparation

A Si wafer (5 mm × 5 mm) was cleaned in an ultra-sonication bath with acetone, methanol and isopropanol in series, before cleaning with oxygen plasma for 5 minutes. The cleaned wafer was glued onto the top surface of a thermal evaporated gold sample previously grown on Si (100 nm thickness) with Epotek 353nd epoxy adhesive to form Si/Glue/Au/Si sandwich structure. The adhesive was cured for 40 minutes at 150 °C, then the original, bottom Si substrate was carefully removed using a sharp blade leaving an atomically-flat Au surface which was templated on the original Si surface.

The prepared gold was scanned by AFM for 3–5 random spots for quality tests. For all cases, only the substrates with roughness below 0.2 nm were used for SAMs growth.

#### SAMs growth

Following the optimised procedure for QCM, the gold was immersed in solution immediately after cleavage without any further treatment for 12 h (molecules **1**, **2** and **4**) and 24 h (molecule **3**). The substrates were rinsed after molecular assembly by THF and ethanol and dried in vacuum for 12 hours (10–2 mbar, 40 °C).

### SAMs characterization

SAM topography was characterized by AFM (MultiMode 8, Bruker Nanoscience) in peak force mode, a low force intermittent-contact mode with combines high resolution imaging, sample nanomechanical information and low sample damage. The peak force setpoint was set to the range of 500 pN to 1 nN and the scan rate was set to 1 Hz. The nano-scratching was performed in contact mode at high set force (*F* = 15–40 nN) using a soft probe (Multi-75-G, *k* = 3 N m^–1^) to ‘sweep away’ the molecular film from a defined area (*A* = 300 nm × 300 nm). The topography of sample after scratching was again characterized in peak force mode, the scratched window is easily observed. Nano-scratching was also conducted on a bare gold sample under the same conditions to ensure no gold is scratched away in used force range. The height difference between the scratched part and un-scratched part indicates the thickness of SAMs.

### Conductive AFM (cAFM)

The electrical transport properties of the SAMs were characterized by a custom cAFM system. The cAFM setup is based on a multi-mode8 AFM system (Bruker nanoscience). The bottom gold substrate was used as the source, and a Pt/Cr coated probe (Multi75 E, BugetSensor) was used as the drain. The force between probe and molecule was controlled at 2 nN, as this force is strong enough for the probe to penetrate through the water layer on the sample surface but not too strong to destroy the molecular thin film. The driven bias was added between the source and drain by a voltage generator (Aglient 33500B), the source to drain current was amplified by a current pre-amplifier (SR570, Stanford Research Systems), and the IV characteristics of the sample was collected by the computer.

### Thermal-electrical atomic force microscopy (ThEFM)

The Seebeck coefficients of SAMs were obtained by a ThEFM modified from the cAFM system used for electrical transport measurement. A Peltier stage driven by a voltage generator (Aglient 33500B, voltage amplified by a wide band amplifier) was used to heat up and cool, thus a temperature difference can be created between sample and probe. The sample temperature was measured by a Type T thermal couple, and the probe temperature was calibrated by using an SThM (scanning thermal microscopy) probe (KNT SThM 2an) under the same conditions (*F* = 2 nN). We made an assumption that the SThM probe and the cAFM probe have similar probe temperatures at the apex part when finding contact with the molecules. The thermal voltage between sample and probe was amplified by high impedance differential pre-amplifier (SR551, Stanford Research Systems), and recorded by a computer.

### Computational details

The ground state Hamiltonian and optimized geometry of each molecule was obtained using the density functional theory (DFT) code SIESTA.^46^ The local density approximation (LDA) exchange correlation functional was used along with double zeta polarized (DZP) basis sets and the norm conserving pseudo potentials. The real space grid was defined by a plane wave cut-off of 250 Ry. The geometry optimization was carried out to a force tolerance of 0.01 eV Å^–1^. This process was repeated for a unit cell with the molecule between gold electrodes where the optimized distance between Au and the pyridine anchor group was found to be 2.3 Å, whereas Au and SMe 2.7 Å. From the ground state Hamiltonian, the transmission coefficient, the room temperature electrical conductance G and Seebeck coefficient S was obtained, as described in Section 2.4, 2.8 and 2.9 in the ESI.† As mentioned above, an earlier study^17^ has shown that the *T*(*E*) for a SAM is approximately the same as that of a single molecule and therefore all calculations were performed on single molecules.

## Author information

Correspondence and requests for materials should be addressed to C. L., L. C., N. L. A. I. and B. R.

## Conflicts of interest

There are no conflicts to declare.

## Supplementary Material

Supplementary information
