# Supplementary material for: Tuning the thermoelectrical properties of anthracene-based self-assembled monolayers
Source: Chem Sci. 2020 Jun 22;11(26):6836–41. doi: 10.1039/d0sc02193h (PMC7504895; doi:10.1039/d0sc02193h)
Supplement: Supplementary file 1 [file SC-011-D0SC02193H-s001.pdf]

Electronic Supplementary Material (ESI) for Chemical Science.
This journal is © The Royal Society of Chemistry 2020

Supplementary Information

Tuning the thermoelectrical properties of anthracene-based self-assembled monolayers

Ali Ismael[a,b,†], Xintai Wang[a,†], Troy L. R. Bennett[c,†], Luke A. Wilkinson[c], Benjamin J. Robinson[a], Nicholas J. Long[c], Lesley F. Cohen[d] and Colin J. Lambert[a]

[a]Physics Department, Lancaster University, Lancaster, LA1 4YB, UK.

[b]Department of Physics, College of Education for Pure Science, Tikrit University, Tikrit, Iraq.

[c]Department of Chemistry, Imperial College London, MSRH, White City, London, W12 0BZ, UK.

[d]The Blackett Laboratory, Imperial College London, South Kensington Campus, London, SW7 2AZ, UK.

†These authors contributed equally to this work

*To whom correspondence should be addressed. e-mail: c.lambert@lancaster.ac.uk; l.cohen@imperial.ac.uk; n.long@imperial.ac.uk; k.ismael@lancaster.ac.uk; b.j.robinson@lancaster.ac.uk

1. Synthesis and characterisation

1.1 Materials and Methods

All reactions were performed with the use of standard air-sensitive chemistry and Schlenk line techniques, under an atmosphere of nitrogen. No special precautions were taken to exclude air during any work-ups. All commercially available reagents were used as received from suppliers, without further purification. 4-Ethynylthioanisole and 4-(ethynyl)phenyl-*tert*-butylthioether were synthesised through adapted literature procedures.[1,2] Solvents used in reactions were collected from solvent towers sparged with nitrogen and dried with 3 Å molecular sieves, apart from DIPA, which was distilled onto activated 3 Å molecular sieves.

1.2 Instrumentation

^{1}H and $^{13}C\{^{1}H\}$ NMR spectra were recorded with a Bruker Avance 400 MHz spectrometer and referenced to the residual solvent peaks of either $CDCl_3$ at 7.26 and 77.2 ppm, respectively. Coupling constants are measured in Hz. Mass spectrometry analyses were conducted by Dr. Lisa Haigh of the Mass Spectrometry Service, Imperial College London. Infrared spectra were recorded on a PerkinElmer Spectrum FT-IR spectrometer

1.3 Synthesis

General procedure for the coupling of terminal alkynes to bromoanthracenes – specific details regarding molar equivalents and column conditions are reported below.

This synthetic procedure is adapted from a published method for Sonogashira coupling.[3] A Schlenk tube was charged with dibromoanthracene, terminal alkyne, CuI (5 mol%) and $Pd(P\text{-}^{t}Bu_3)_2$ (5 mol%) then placed under an inert atmosphere. Anhydrous DIPA and toluene were added to the reaction vessel via cannula and the reaction was stirred overnight at room temperature to generate a bright orange/yellow precipitate. Removal of the solvent *in vacuo* led to a dark brown crude material which can be purified by column chromatography.

9,10-Di(4-(ethynyl)pyridine)anthracene (1)[6]

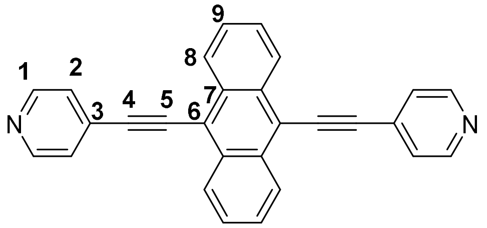

4-(Ethynyl)pyridine hydrochloride (0.13 g, 0.94 mmol), 9,10-dibromoanthracene (0.15 g, 0.47 mmol), CuI (0.01 g, 0.02 mmol) and $Pd(P\text{-}^{t}Bu_3)_2$ (0.01 g, 0.02 mmol) gave an orange-brown solid which was purified by chromatography on an alumina (grade V) column, eluted with DCM to give the product as an orange solid (0.08 g, 0.22 mmol, 46%).

^{1}H NMR ($CDCl_3$, 298 K, 400 MHz): δ = 8.73 (d, $^{3}J_{H\text{-}H}$ = 5.6 Hz, 4H, *H1*), 8.69-8.63 (m, 4H, *H9*), 7.74-7.68 (m, 4H, *H8*), 7.63 (d, $^{3}J_{H\text{-}H}$ = 5.6 Hz, 4H, *H2*) ppm; **$^{13}C\{^{1}H\}$ NMR** ($CDCl_3$, 298 K, 100 MHz): δ = 150.2 (Ar-C-H), 132.4 (Ar-C-C), 131.4 (Ar-C-C), 127.6 (Ar-C-H), 127.2 (Ar-C-H), 125.7 (Ar-C-H), 118.3 (Ar-C-C), 99.8 (-C≡C-), 90.9 (-C≡C-) ppm; **IR**: 2201 (-C≡C-) cm^{-1}; **MS** ES+: calcd. for $C_{28}H_{16}N_2$ [M]+ 381.1388; found. 381.1392.

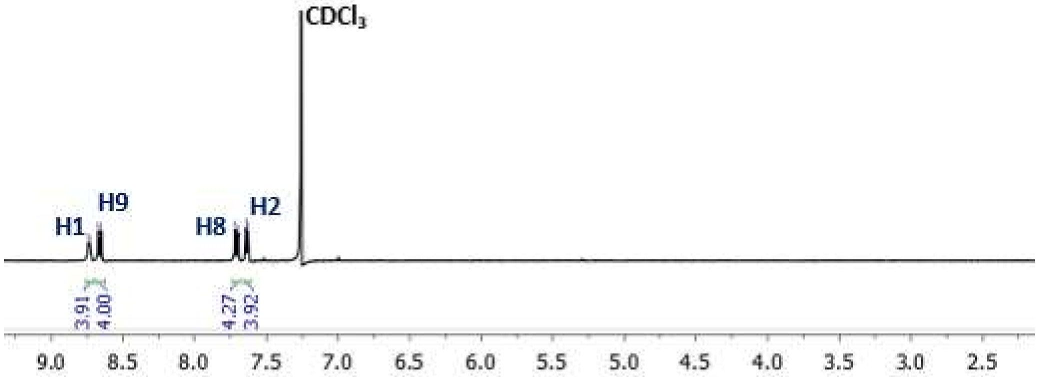

Figure S1: The ^{1}H NMR spectrum of **1** in $CDCl_3$

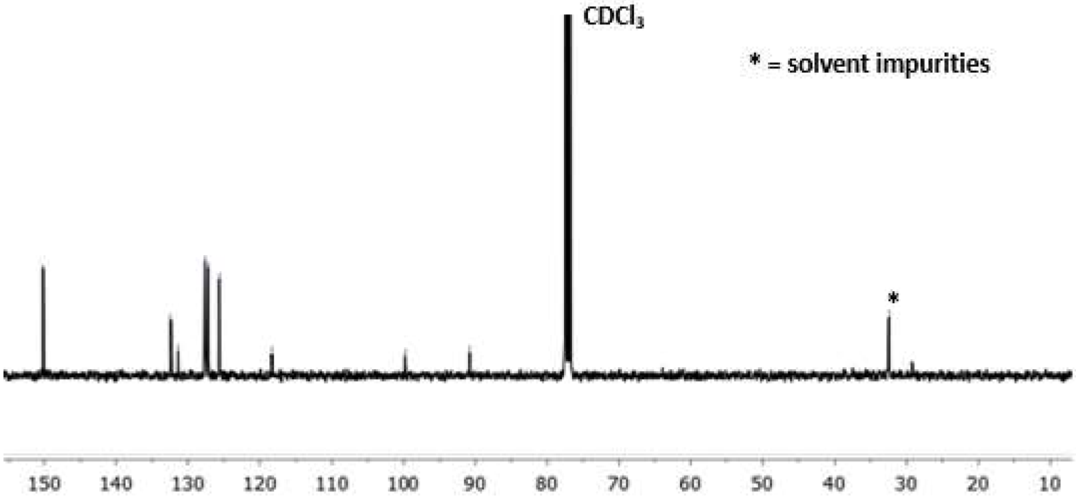

Figure S2: The ^{13}C{^{1}H} NMR spectrum of **1** in $CDCl_3$

9-Bromo-10-(4-(ethynyl)thioanisole)anthracene (2A)

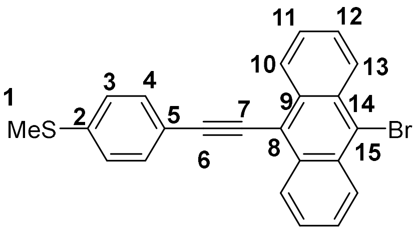

4-(Ethynyl)thioanisole (0.15 g, 1.01 mmol), 9,10-dibromoanthracene (1.70 g, 5.06 mmol), CuI (0.01 g, 0.05 mmol) and Pd(P-[t]$Bu_3)_2$ (0.03 g, 0.05 mmol) gave an orange-brown solid which was purified by chromatography on a silica column, eluted with n-hexane/DCM (1:0 → 4:1 v/v) to give the product as a yellow solid (0.10 g, 0.25 mmol, 25%).

^{1}H NMR ($CDCl_3$, 298 K, 400 MHz): δ = 8.71-8.66 (m, 2H, *H10*), 8.61-8.56 (m, 2H, *H13*), 7.68 (dd, $^3J_{H\text{-}H}$ = 6.8, $^4J_{H\text{-}H}$ = 2.0 Hz, 2H, *H4*), 7.67-7.60 (m, 4H, *H11, H12*), 7.32 (dd, $^3J_{H\text{-}H}$ = 6.8, $^4J_{H\text{-}H}$ = 2.0 Hz, 2H, *H3*), 2.55 (s, 3H, *H1*) ppm; **^{13}C{^{1}H} NMR** ($CDCl_3$, 298 K, 100 MHz): δ = 140.2 (Ar-C-C), 133.1 (Ar-C-C), 132.0 (Ar-C-H), 130.4 (Ar-C-C), 128.4 (Ar-C-H), 127.6 (Ar-C-C), 127.4 (Ar-C-H), 126.9 (Ar-C-H), 126.1 (Ar-C-H), 124.2 (Ar-C-C) 119.7 (Ar-C-C), 118.5 (Ar-C-C), 101.9 (-C≡C-), 86.3 (-C≡C-), 15.6 ($S\text{-}CH_3$) ppm; **IR**: 2147 (-C≡C-) cm^{-1}; **MS** ES+: calcd. for $C_{23}H_{15}BrS$ [M]+ 403.0151; found. 403.0149.

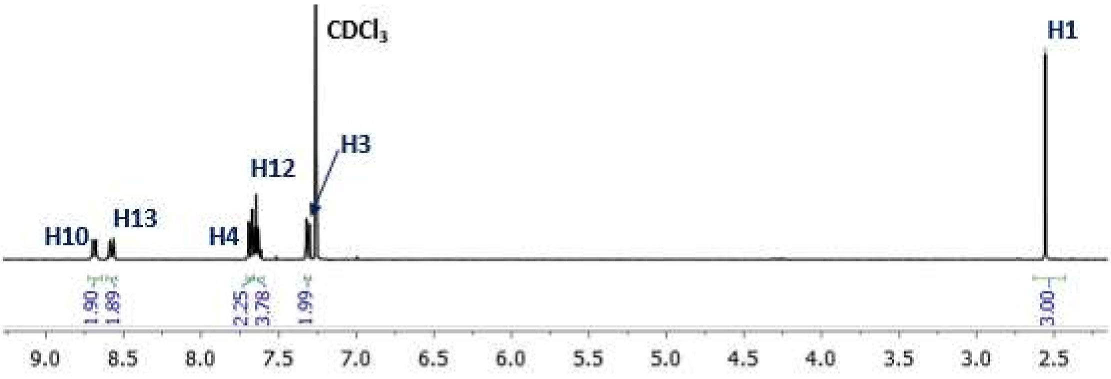

Figure S3: The ^{1}H NMR spectrum of **2A** in $CDCl_3$

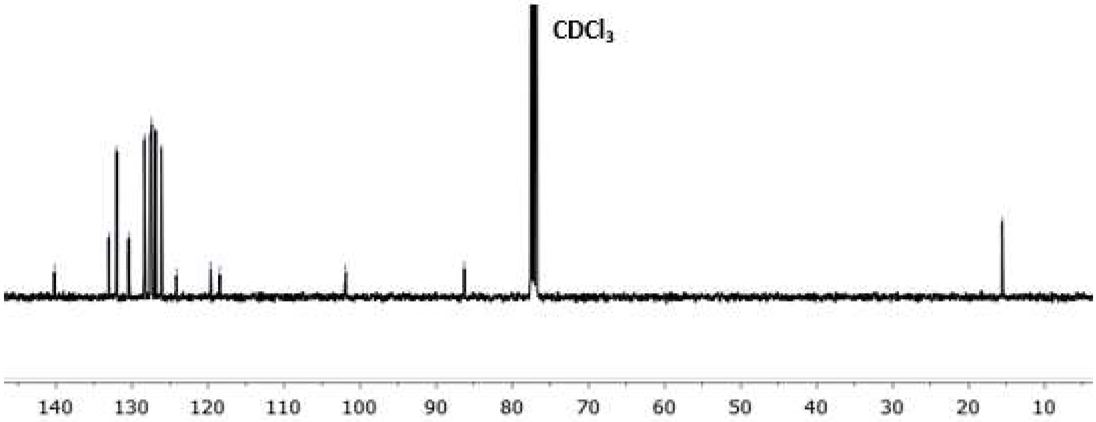

Figure S4: The $^{13}C\{^1H\}$ NMR spectrum of **2A** in $CDCl_3$

9-(4-(Ethynyl)pyridine)-10-(4-(ethynyl)thioanisole)anthracene (2)

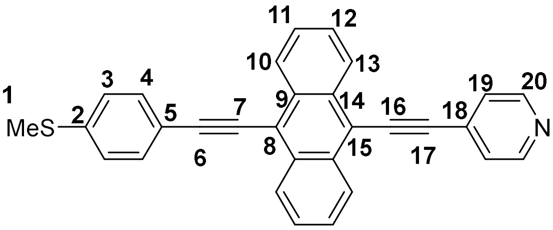

4-(Ethynyl)pyridine hydrochloride (0.04 g, 0.30 mmol), (**2A**) (0.12 g, 0.30 mmol), CuI (0.01 g, 0.07 mmol) and Pd(P-^{t}Bu$_3$)$_2$ (0.03 g, 0.07 mmol) gave an orange-brown solid which was purified by chromatography on a silica column, eluted with n-hexane/THF (1:0 → 1:1 v/v) to give the product as an orange solid (0.07 g, 0.16 mmol, 53%).

^{1}H NMR ($CDCl_3$, 298 K, 400 MHz): δ = 8.75-8.65 (m, 6H, *H11, H12, H20*), 7.75-7.67 (m, 6H, *H10, H13, H19*), 7.64 (dd, $^3J_{H-H}$ = 4.4, $^4J_{H-H}$ = 1.6 Hz, 2H, *H4*), 7.34 (dd, $^3J_{H-H}$ = 4.4, $^4J_{H-H}$ = 1.6 Hz, 2H, *H3*), 2.56 (s, 3H, *H1*) ppm; **$^{13}C\{^1H\}$ NMR** ($CDCl_3$, 298 K, 100 MHz): δ = 150.1 (Ar-C-H), 140.5 (Ar-C-C), 132.5 (Ar-C-C), 132.1 (Ar-C-H), 132.0 (Ar-C-C), 131.6 (Ar-C-C), 127.6 (Ar-C-H), 127.5 (Ar-C-H), 127.0 (Ar-C-H), 127.0 (Ar-C-H), 126.1 (Ar-C-H), 125.6 (Ar-C-H), 120.2 (Ar-C-C), 119.5 (Ar-C-C), 116.7 (Ar-C-C), 103.2 (-C≡C-), 99.3 (-C≡C-), 91.2 (-C≡C-), 86.6 (-C≡C-), 15.5 (S-CH_3) ppm; **IR**: 2187 (-C≡C-) cm^{-1}; **MS** ES+: calcd. for $C_{30}H_{19}NS$[M]+ 426.1311; found. 426.1310.

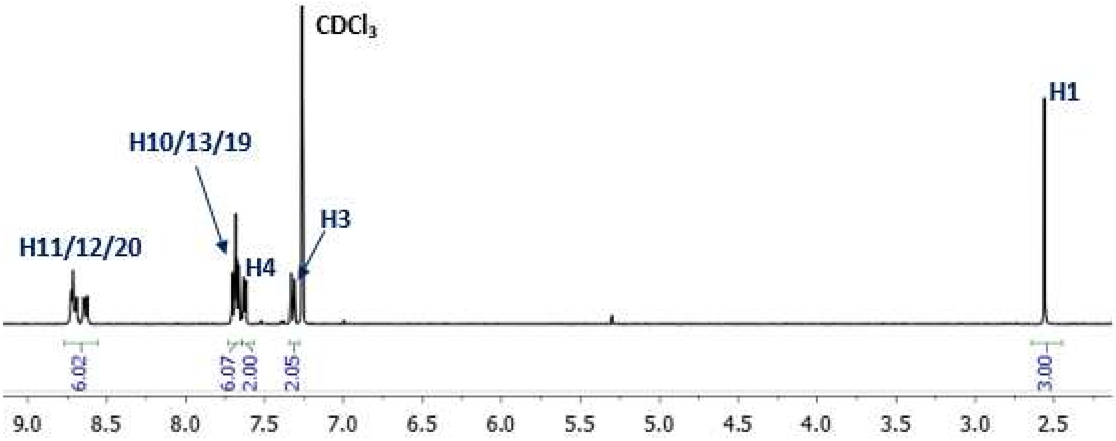

Figure S5: The ^{1}H NMR spectrum of **2** in $CDCl_3$

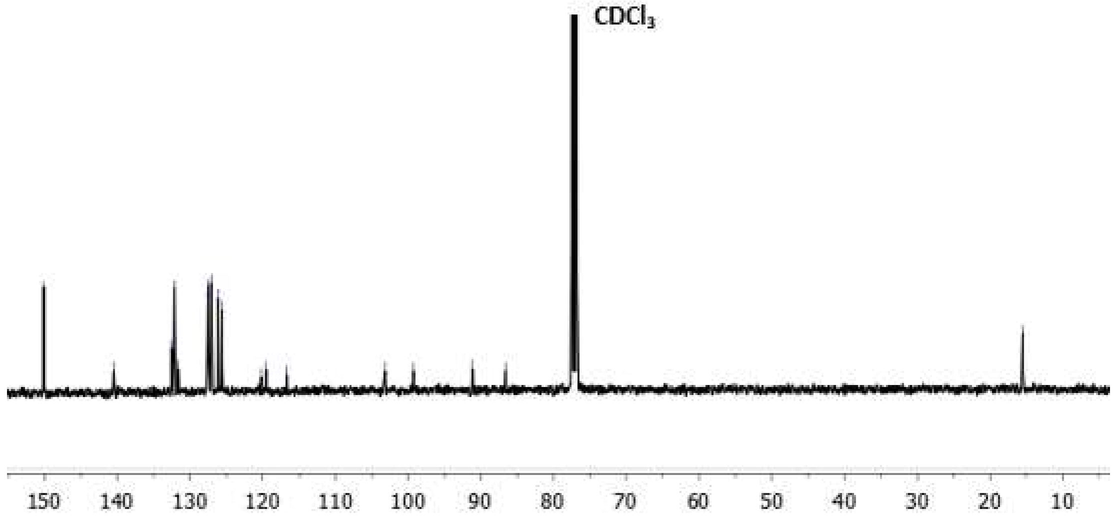

Figure S6: The ^{13}C NMR spectrum of **2** in $CDCl_3$

9,10-Di(4-(ethynyl)thioanisole)anthracene (3)[5]

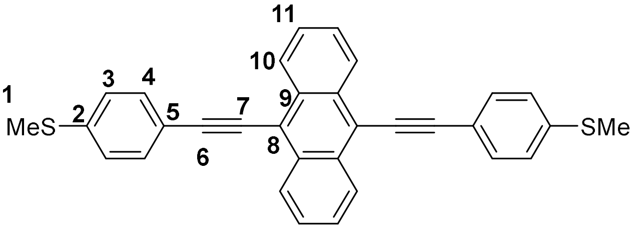

4-(Ethynyl)thioanisole (0.20 g, 1.35 mmol), 9,10-dibromoanthracene (1.81 g, 5.40 mmol), CuI (0.01 g, 0.07 mmol) and Pd(P-^{t}Bu$_3$)$_2$ (0.03 g, 0.07 mmol) gave an orange-brown solid which was purified by chromatography on a silica column, eluted with n-hexane/THF (1:0 → 1:1 v/v) to give the product as an orange solid (0.20 g, 0.85 mmol, 63%).

^{1}H NMR ($CDCl_3$, 298 K, 400 MHz): δ_H = 8.69 (dd, $^3J_{H-H}$ = 6.8, $^4J_{H-H}$ = 3.2 Hz, 4H, *H10*), 7.70 (d, $^3J_{H-H}$ = 8.4 Hz, 4H, *H4*), 7.67 (dd, $^3J_{H-H}$ = 6.8, $^4J_{H-H}$ = 3.2 Hz, 4H, *H11*), 7.32 (d, $^3J_{H-H}$ = 8.4 Hz, 4H, *H3*), 2.55 (s, 6H, *H1*) ppm; **^{13}C {^{1}H} NMR** ($CDCl_3$, 298 K, 100 MHz): δ_C = 140.2 (Ar-C-C), 132.2 (Ar-C-C), 132.1 (Ar-C-H), 127.4 (Ar-C-H), 126.9 (Ar-C-

H), 126.2 (Ar-C-H), 119.8 (Ar-C-C), 118.6 (Ar-C-C), 102.5 (-C≡C-), 86.9 (-C≡C-), 15.6 (S-CH_3) ppm; **IR**: 2195 (-C≡C-) cm^{-1}; **MS** ES+: calcd. for $C_{32}H_{22}S_2$ [M]+ 469.1079; found. 469.1077.

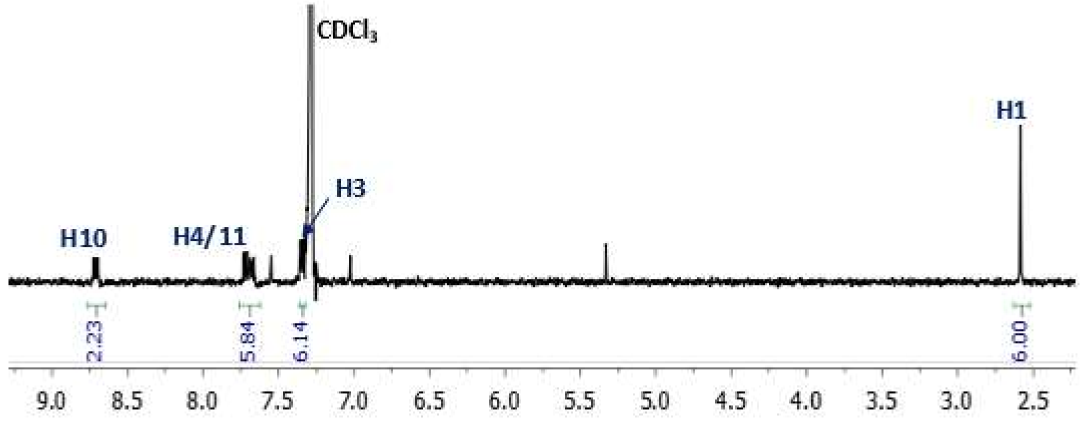

Figure S7: The ^{1}H NMR spectrum of **3** in $CDCl_3$

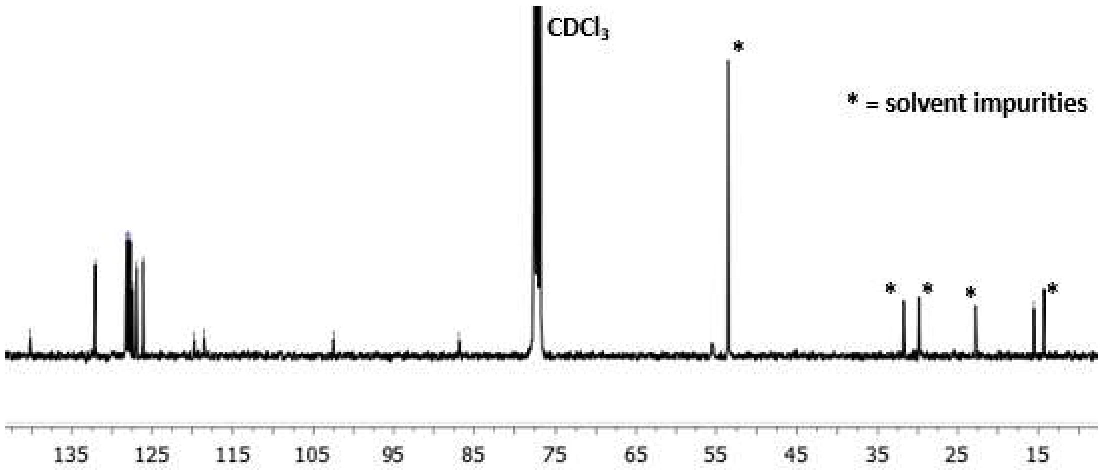

Figure S8: The $^{13}C\{^1H\}$ NMR spectrum of **3** in $CDCl_3$

9,10-Di(4-(ethynyl)phenyl-*tert*-butylthioether)anthracene (4A)[4]

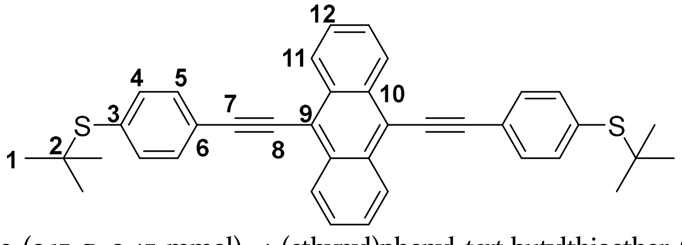

9,10-Dibromoanthracene (0.15 g, 0.45 mmol), 4-(ethynyl)phenyl-*tert*-butylthioether (0.21 g, 1.12 mmol), CuI (0.01 g, 0.05 mmol) and Pd(P-^{t}Bu_3)$_2$ (0.02 g, 0.05 mmol) gave an orange-brown solid which was purified by chromatography on a silica column, eluting with n-hexane/DCM (1:0→8:2) to give the product as an orange solid (0.19 g, 0.34 mmol, 76%).

^{1}H NMR ($CDCl_3$, 298 K, 400 MHz): δ_H = 8.72-8.67 (m, 4H, *H*11), 7.74 (d, $^3J_{H\text{-}H}$ = 8.4 Hz, 4H, *H*5), 7.68-7.63 (m, 4H, *H*12), 7.63 (d, $^3J_{H\text{-}H}$ = 8.4 Hz, 4H, *H*4), 1.35 (s, 18H, *H*1) ppm; **$^{13}C\{^1H\}$ NMR** ($CDCl_3$, 298 K, 100 MHz): δ_C = 137.6 (Ar-C-H), 134.0 (Ar-C-C), 132.3 (Ar-C-C), 131.7 (Ar-C-H), 127.4 (Ar-C-H), 127.1 (Ar-C-H), 123.9 (Ar-C-C), 118.6 (Ar-C-C), 102.1 (-C≡C-), 88.1 (-C≡C-), 46.8 (S-C-C), 31.2 (CH_3) ppm; **IR**: 2194 (-C≡C-) cm^{-1}; **MS** APCI: calcd. [M]+ 555.2175; found. 555.2171.

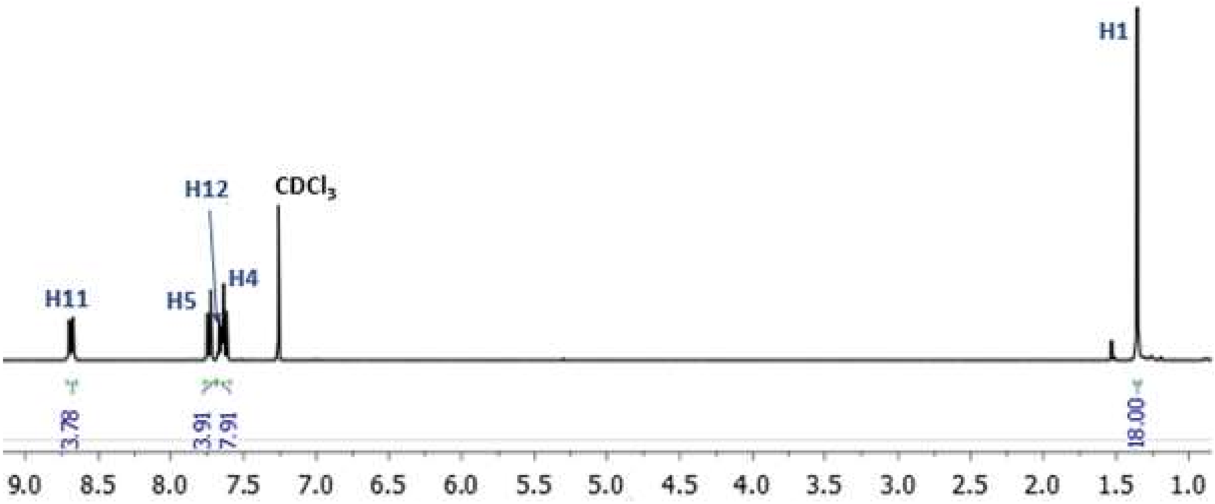

Figure S9: The ^{1}H NMR spectrum of **4A** in $CDCl_3$

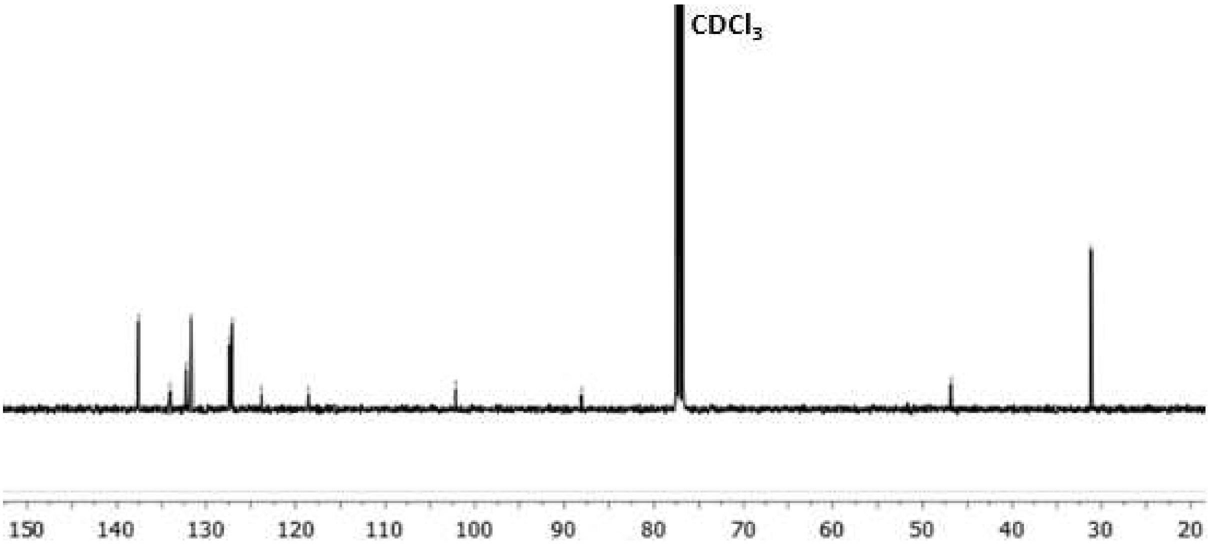

Figure S10: The $^{13}C\{^1H\}$ NMR spectrum of **4A** in $CDCl_3$

9,10- Di(4-(ethynyl)phenylthioacetate)anthracene (4)[4]

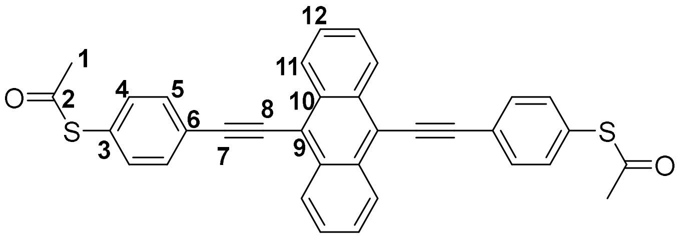

Synthesised according to an adapted literature procedure.[4] (**4A**) (0.08 g, 0.14 mmol) was dissolved in DCM (30 mL) and toluene (30 mL). Acetyl chloride (1 mL) was added and the solution was degassed for 20 minutes. BBr_3 (1 M in hexanes, 0.72 mL, 0.72 mmol) was added and the solution was stirred overnight at room temperature. The solvent was removed *in vacuo* and the crude product was exposed to chromatography on a silica column, eluting with n-hexane/DCM (1:0 → 1:1). The product was washed with hexane (3 x 100 mL) and recrystallized from DCM to yield dark orange crystals (0.03 g, 0.06 mmol, 43%).

^{1}H NMR ($CDCl_3$, 298 K, 400 MHz) δ_H = 8.71-8.64 (m, 4H, *H*11), 7.81 (d, $^3J_{H-H}$ = 8.4 Hz, 4H, *H*5), 7.69-7.62 (m, 4H, *H*12), 7.51 (d, $^3J_{H-H}$ = 8.4 Hz, 4H, *H*4), 2.48 (s, 6H, *H*1) ppm; **^{13}C {^{1}H} NMR** ($CDCl_3$, 298 K, 100 MHz): δ_C = 193.4 (-C=O), 134.4 (Ar-C-H), 132.2 (Ar-C-H), 132.2 (Ar-C-C), 128.6 (Ar-C-C), 127.2 (Ar-C-H), 127.0 (Ar-C-H), 124.6 (Ar-C-C), 118.4 (Ar-C-C), 101.7 (-C≡C-), 88.1 (-C≡C-), 30.4 (CH_3) ppm; **IR**: 2195 (-C≡C-), 1692 (-C=O) cm^{-1}; **MS** ES+: calcd. [M]+ 527.1134; found. 527.1128.

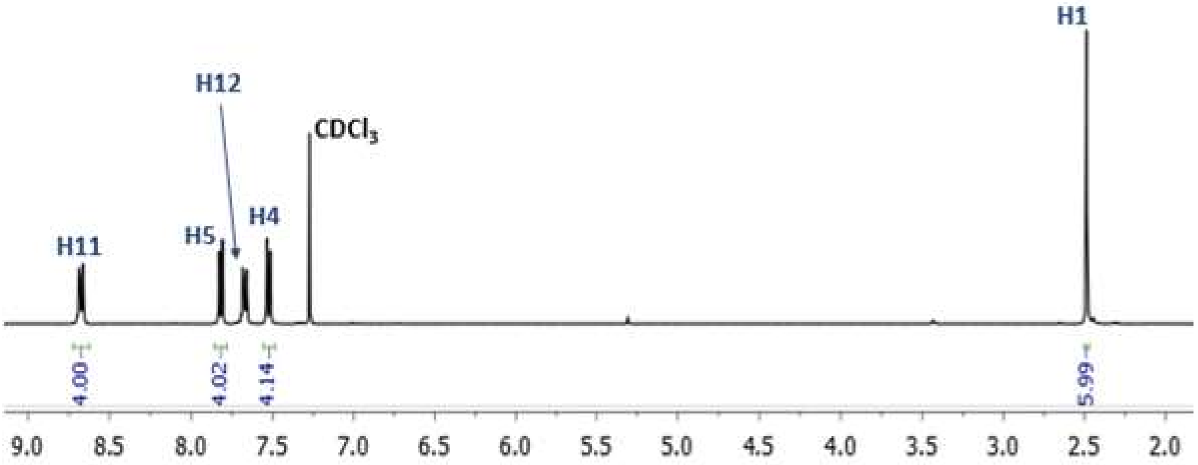

Figure S11: The ^{1}H NMR spectrum of **4** in $CDCl_3$

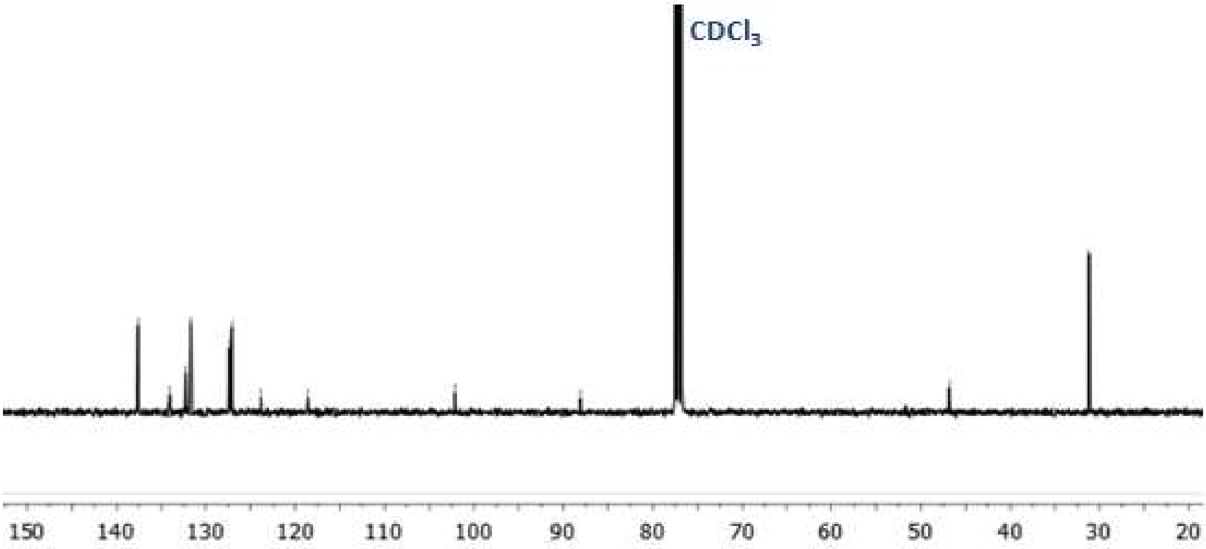

Figure S12: The ^{13}C{^{1}H} NMR spectrum of **4** in $CDCl_3$

2. SAM preparation and characterisation.

2.1 Au preparation

The ultra-flat gold was prepared by modifying the template stripped (TS) method of Whitesides and Pinkhassik on mica (Agar scientific).11 The Si wafer (in 5 mm x 5 mm) was ultra-sonicated in acetone, methanol and isopropanol in succession and then cleaned with oxygen plasma for 5 minutes. The cleaned wafer was glued onto the gold deposited Mica substrate with Epotek 353nd

epoxy adhesive to form Si/Glue/Au/Mica sandwich structure. After 40 minutes curing of glue at 1500C, the ultra-flat TS gold was obtained by eliminating the mica with a knife. The prepared gold was scanned by AFM for 3-5 random spots as a quality test. For all cases, the average roughness of the gold was about 0.1 nm.

2.2 SAM growth

0.1 mM solution with target SAM molecules dissolved in toluene was prepared, with 10 minute deoxygenating by nitrogen bubbling. The freshly cleaved TS-Au without any further treatment was immersed into the solution, and incubated for 24 hours in vacuum.
After SAM growth, the sample was rinsed with toluene, ethanol and isopropanol for several times to remove physisorped molecules. The sample after rinsing was blown with nitrogen for drying, and incubated in vacuum oven (10-2 mbar) overnight at 350C for solvent evaporation.

2.3 QCM measurement

New QCM crystal (5mm diameter, fo = 10MHz, from icryst) was cleaned by oxygen plasma for 10 minutes, immersed in hot DMF (1000C) for 2 hours, and in room temperature DMF overnight, washed with ethanol and isopropanol, and dried in vacuum oven for 20 hours at 350C. The QCM measurement is done by an openQCM system. The initial resonance frequency of the cleaned substrate was recorded, and the substrate was used for SAMs growth. The procedure for SAMs growth was the same as for TS gold. The frequency of QCM crystal after SAMs growth was then recorded. The difference between the frequency before and after SAMs growth, Δf, can be used to determine the occupation area of a single molecule on the substrate via Sauerbrey equation:

$$A_{\text{molecule}} = \frac{A' * Mw}{(k * \Delta f * A * N_A)}$$

$$k = \frac{\sqrt{\mu * \rho}}{2 * f_0^2}$$

Where A’ the electrode area, A the crystal area, Δf the frequency change, Mw the molecular weight, NA the Avogadro’s number, μ the shear modulus of quartz, ρ the density of quartz, fo the initial frequency.

The calculated single molecular occupation area for 2Py is 51 A2, PySMe is 39 A2, 2SMe is 41 A2 , and 2SAc is 34 A2. 12

2.4Seebeck measurement:

The Seebeck in this work is measured by thermoelectric atomic force microscopy (THEAFM). An Pt coated blunt probe was used as the top electrode, and the temperature of the bottom electrode (Au substrate) was controlled by a peltier stage. A Cu-CuNi thermocouple was used to calibrate the sample temperature after the measurement. The probe temperature was estimated by using a KMT thermistor probe (Pd-NiCr coating) with similar radius curvature to the Pt coated probe, and the temperature of the apex part of the thermistor probe was recorded at different sample temperature by a Maxwell bridge. Since the radius curvature of the thermistor probe was similar with the probe we use, we assume the apex temperature was similar in these two cases. The temperature difference between sample and probe was defined as ΔT. The voltage between sample and probe, VTherm, was extracted by a voltage pre-amplifier (SRS560) with a low pass filter, and recorded by the computer. The laser deflection of probe shifted a bit during temperature change due to thermal expansion, and we set it to a constant value manually by regulating the setpoint. The Seebeck coefficient was obtained from equation:

$$S_{junction} = S_{probe-substra} \quad - \frac{\Delta V_{Therm}}{\Delta T}$$

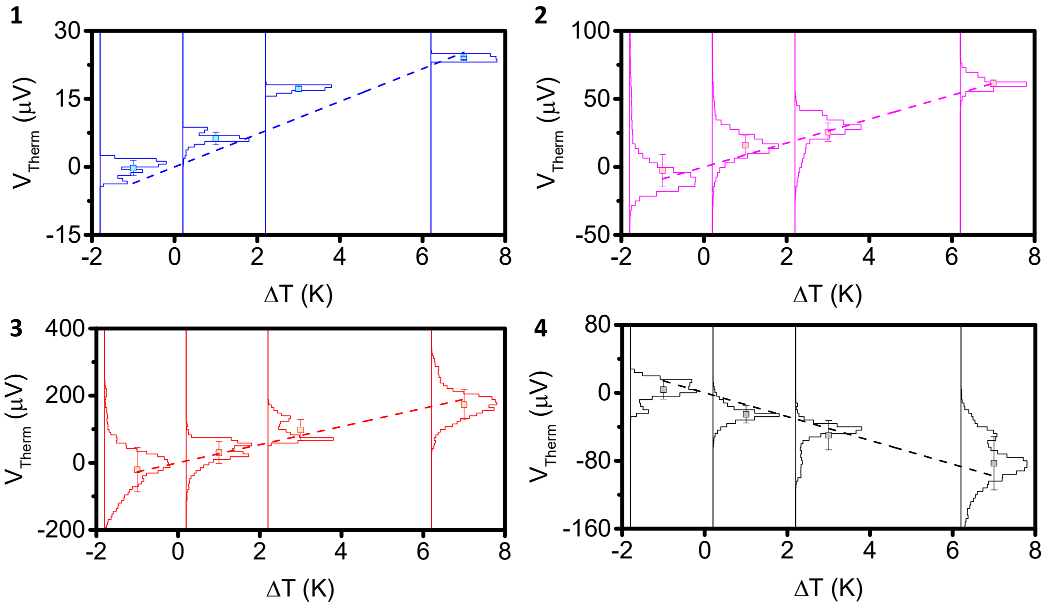

Figure S13: Plots of thermal voltage vs. temperature difference of SAMs **1-4** in histogram and linear fits to their averages **1-4**.

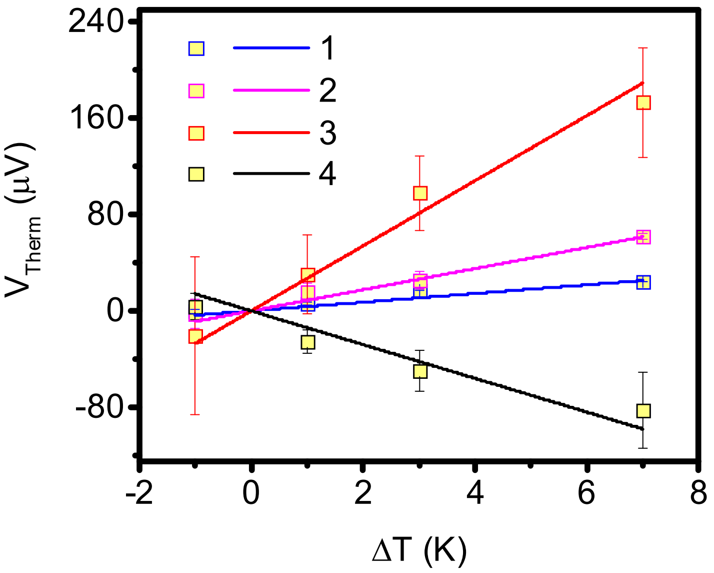

Figure S14: Plots of average thermal voltages vs. temperature difference of SAMs **1-4**.

Table S1: Surface properties of molecular thin film formed by different molecules

Molecules	Area by QCM (m²)	Thickness (nm)	Roughness （nm）
1	5.10E-19	1.12	0.43
2	3.90E-19	1.19	0.09
3	4.10E-19	1.25	0.17
4	3.40E-19	1.12	0.15

2.5 cAFM characterisation

The electrical characterization in this work was obtained by conductive AFM. The Pt coated probe was used as the top contact. A sweeping bias voltage from a function generator was applied between the gold and probe for current flow. The current signal between sample and probe was amplified by an IV converter (DLPCA200, Femto) and recorded by a computer.

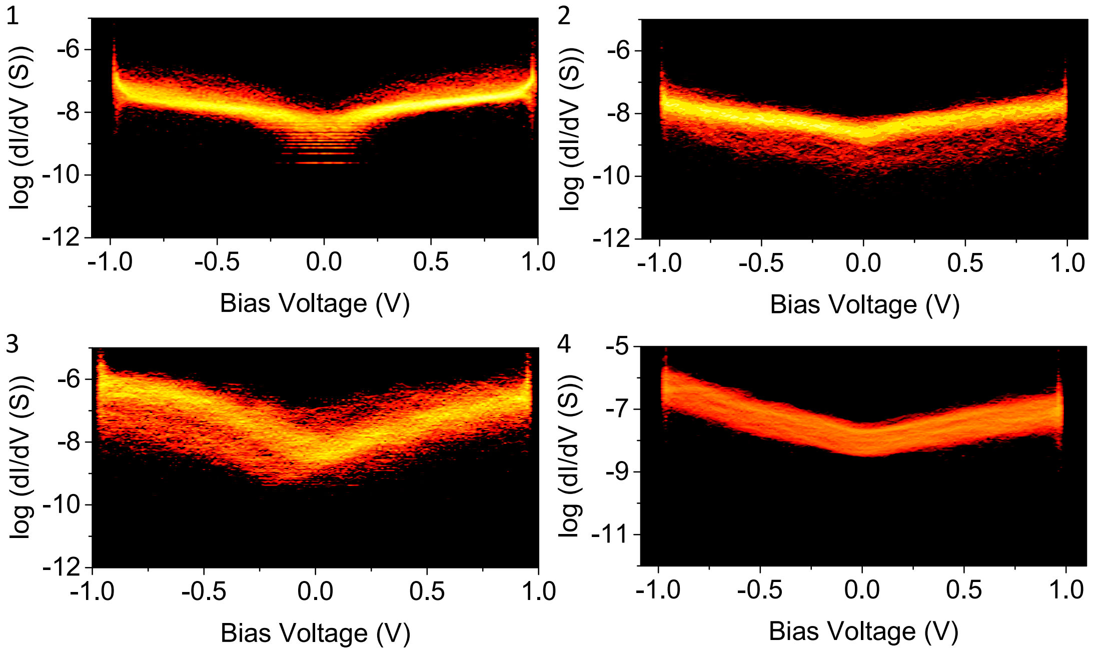

Figure S15: The distribution of differential conductance of SAMs 1-4.

The force between sample and probe was controlled by the laser deflection and fixed at 2nN. The contact area between sample and probe was estimated via Johnson-Kendall-Roberts (JKR) contact model. The contact radius, r, was calculated via equation:

$$r = (F \times R \times \frac{1}{Y})^{\frac{1}{3}}$$

$$\frac{1}{Y} = \frac{3}{4} \times (\frac{1 - v_1^2}{E_1} + \frac{1 - v_2^2}{E_2})$$

r the contact radius, F the loading force from probe to sample (2nN), R the radius of the probe, v1 and v2 the Poisson ratio of the material, E1 and E2 the Young's Modulus for probe and SAMs.

The radius of the probe was obtained from SEM image, and estimated to be 25 nm. The Young's modulus was obtained from AFM in peakforce mode, which was about 2 GPa for all the SAMs. Other parameters were obtained from literature working on similar systems.

2.6 SAM quality

The SAMs sample on TS gold was measured by AFM (multi-mode 8, Brucker) in peak force mode. The roughness of the sample surface was obtained by nano-scope 9.0 software. The SAMs thickness was obtained by nano-scratching. A small region of molecular thin film was scratched by a stiff AFM probe at high force (50 nN), and the peak force mode was used to scan a large region. The scratched window can be observed at the large scale scan, and the height difference indicates thickness of the film as shown in Figure s16. The measured height and the corresponding tilting angle was listed in Table S2.

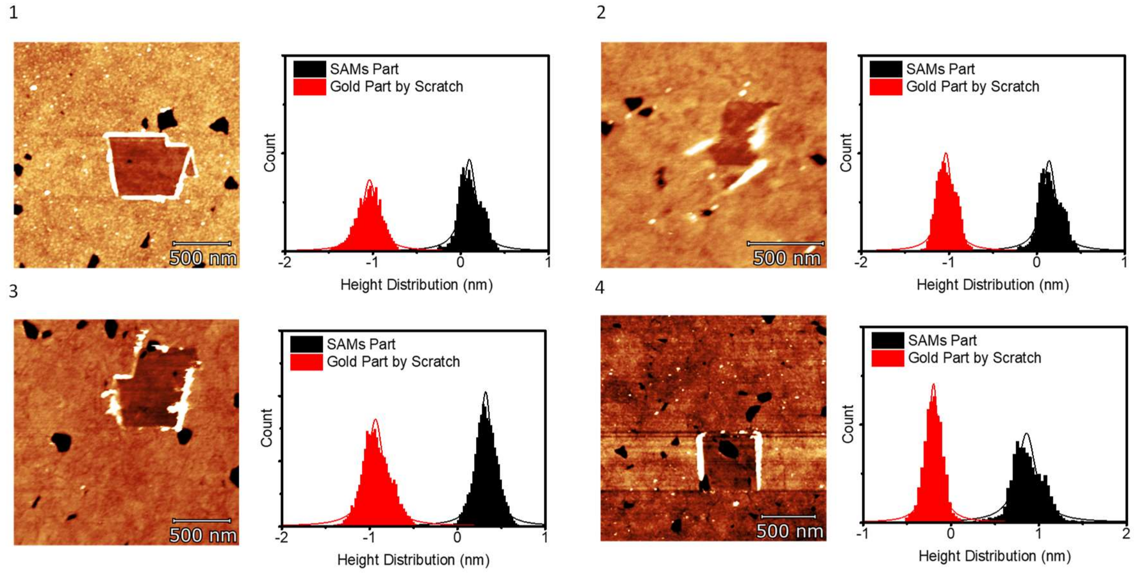

Figure S16: The surface topography with nanoscratching and the height distribution of SAMs 1-4 characterized by AFM.

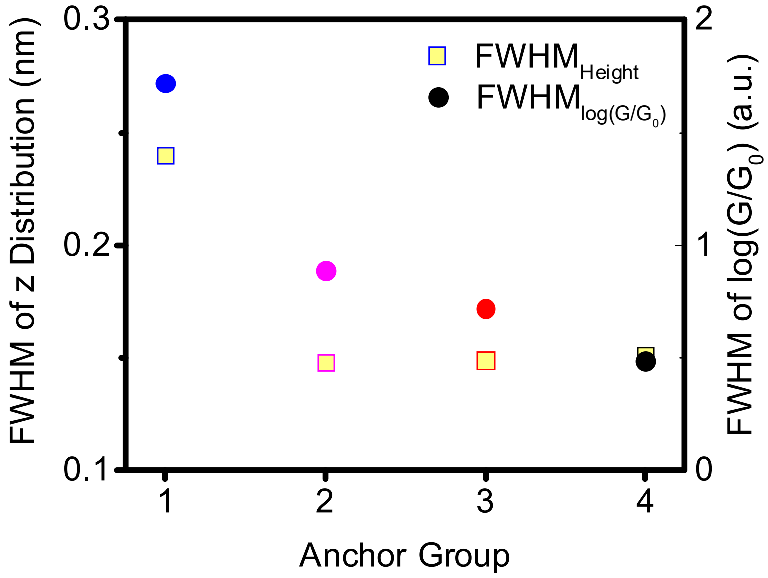

Figure S17: FWHM of conductance (circles) and height (squares) distributions, obtained from Gaussian fits to the histograms in Figure 3, for each anchor group.

3. Additional DFT and Transport Calculations

3.1 Optimised DFT Structures of Isolated Molecules

Using the density functional code SIESTA,[7,8] the optimum geometries of the isolated molecules **1-4** were obtained by relaxing the molecules until all forces on the atoms were less than 0.01 eV / Å as shown in Figure S18. A double-zeta plus polarization orbital basis set, norm-conserving pseudopotentials, an energy cut-off of 250 Rydbergs defined the real space grid were used and the local density approximation (LDA) was chosen to be the exchange correlation functional. We also computed results using GGA and found that the resulting transmission functions were comparable with those obtained using LDA.[9-11]

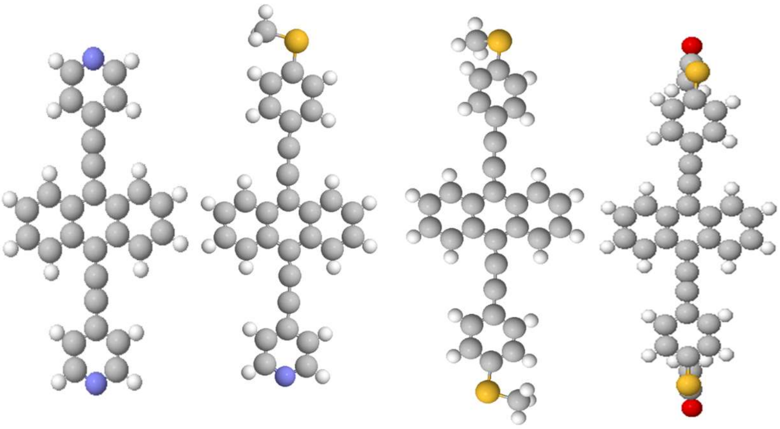

1 (2Py) 2 (PySMe) 3 (2SMe) 4 (2SAc)

Figure S18: Fully relaxed isolated molecules. Key: C = grey, H = white, N = blue, S = yellow, O = red.

3.2 Frontier orbitals of the molecules

The plots below show isosurfaces of the HOMO, LUMO, HOMO-1 and LUMO+1 of isolated molecules 1-4.

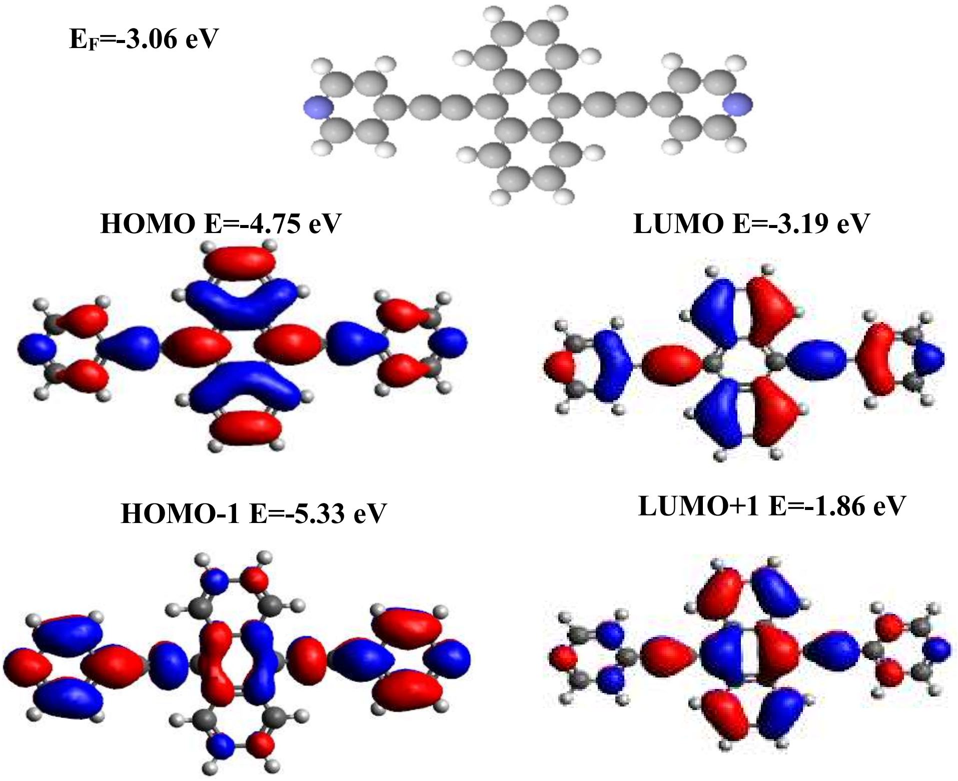

Figure S19: Wave function for 1. Top panel: Fully optimised geometry of 1. Lower panel: HOMO, LUMO, HOMO-1 and LUMO+1 along with their energies

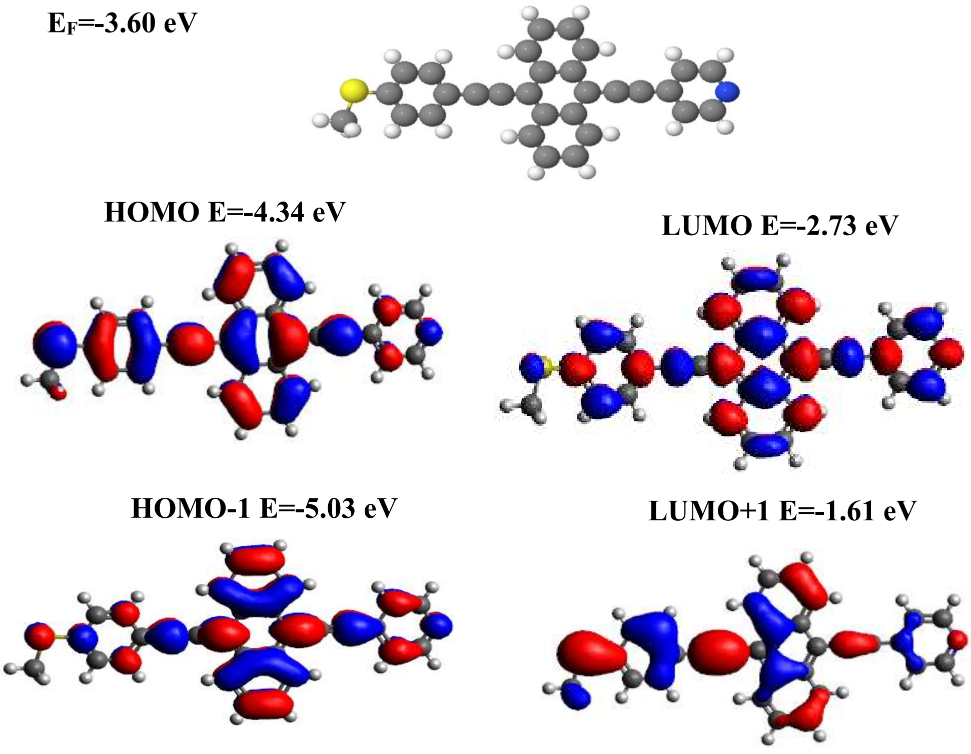

Figure S20: Wave function for **2**. Top panel: Fully optimised geometry of **2**. Lower panel: HOMO, LUMO, HOMO-1 and LUMO+1 along with their energies

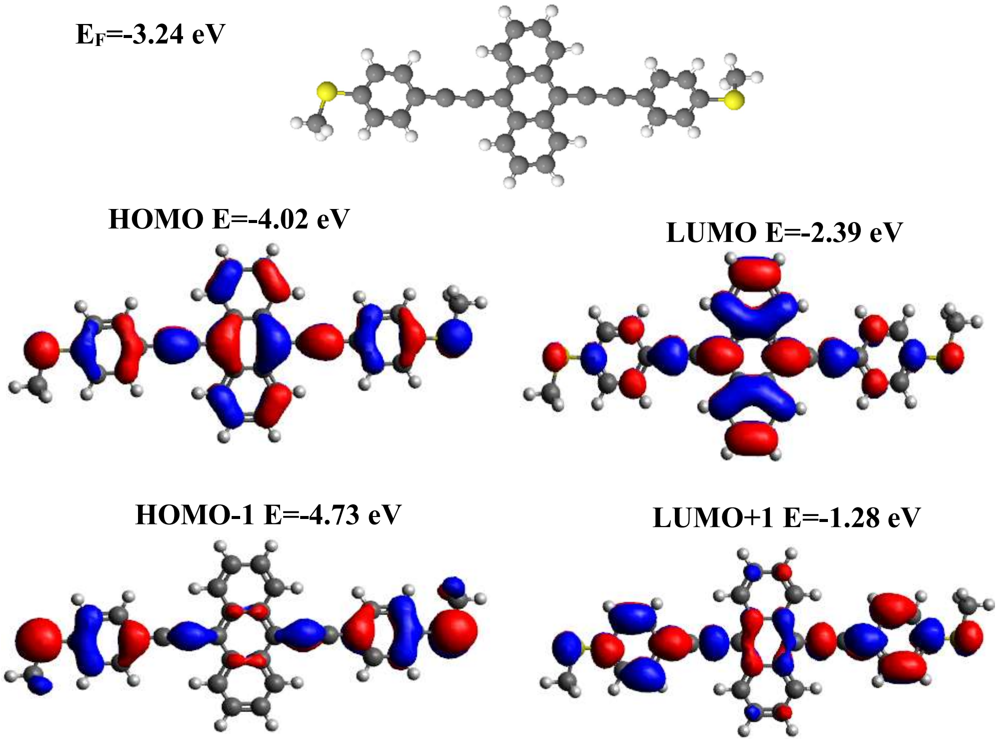

Figure S21: Wave function for **3**. Top panel: Fully optimsed geometry of **3**. Lower panel: HOMO, LUMO, HOMO-1 and LUMO+1 along with their energies

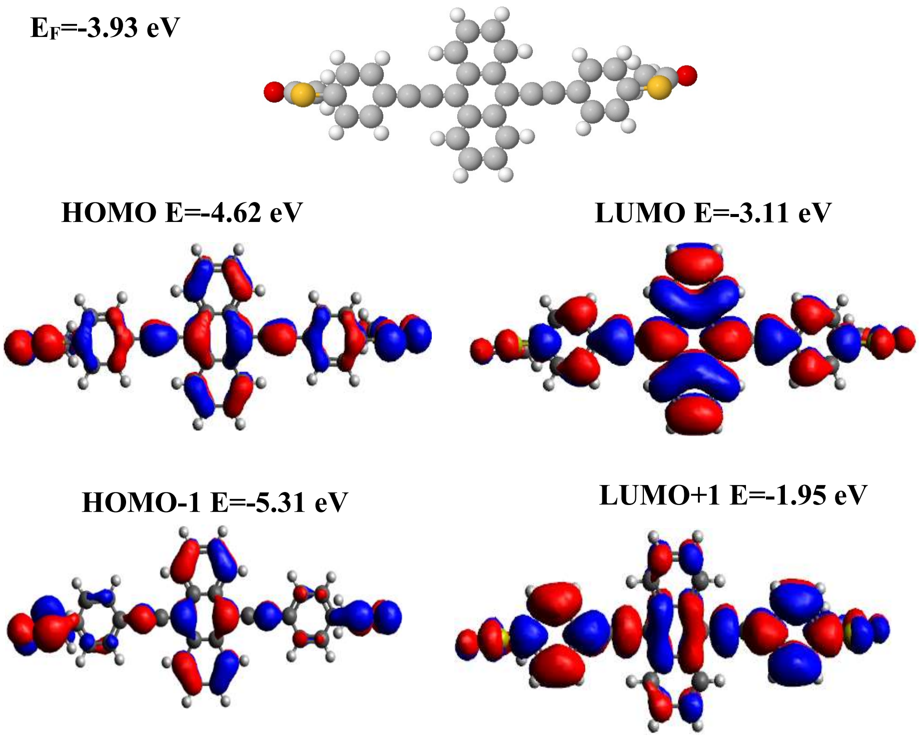

Figure S22: Wave function for **4**. Top panel: Fully optimsed geometry of **4**. Lower panel: HOMO, LUMO, HOMO-1 and LUMO+1 along with their energies

3.3 Product rule

Wave function plots for isolated molecules with their optimised geometries (Figures S14-S17) show iso-surfaces of the HOMO, LUMO, HOMO-1 and LUMO+1 of isolated molecules of the studied molecules. The orbital product rule[12-14] predicts CQI in the HOMO-LUMO gap for the molecules of study, because from Figures S19-S22, the product of the HOMO (LUMO) amplitudes at opposite ends of the molecules are negative (positive). Table S2 summarises the signs of these orbital products.

Table S2: Product rule predictions of the studied molecules, (c= constructive, d=destructive, blue= -ive and red= +ive).

Compound	H-1	H	L	L+1	G_{H-L}
**1**<br>**E (eV)**	-<br>**-5.33**	+<br>**-4.75**	-<br>**-3.19**	-<br>**-1.865**	**c**
**2**<br>**E (eV)**	-<br>**-5.03**	+<br>**-4.34**	-<br>**-2.73**	+<br>**-1.61**	**c**
**3**<br>**E (eV)**	+<br>**-4.73**	-<br>**-4.02**	+<br>**-2.39**	-<br>**-1.28**	**c**
**4**<br>**E (eV)**	+<br>**-5.31**	-<br>**-4.62**	+<br>**-3.11**	-<br>**-1.95**	**c**

3.4 Binding energy of molecules on Au

To calculate the optimum binding distance between pyridyl/methyl sulphide anchor groups and Au(111) surfaces, we used DFT and the counterpoise method, which removes basis set superposition errors (BSSE). The binding distance d is defined as the distance between the gold surface and the N/SMe terminus of the pyridyl/methyl sulphide group. Here, compound **4** is defined as entity A and the gold electrode as entity B. The ground state energy of the total system is calculated using SIESTA and is denoted E_{AB}^{AB}. The energy of each entity is then calculated in a fixed basis, which is achieved using ghost atoms in SIESTA. Hence, the energy of the individual **4** in the presence of the fixed basis is defined as E_{A}^{AB} and for the gold as E_{B}^{AB}. The binding energy is then calculated using the following equation:

$$\text{Binding Energy} = E_{AB}^{AB} - E_{A}^{AB} - E_{B}^{AB} \qquad \text{(S1)}$$

We then considered the nature of the binding depending on the gold surface structure. We calculated the binding to a Au pyramid on a (111) surface with the nitrogen/methyl sulphide atom binding at a 'top' site and then varied the binding distance d. Figure S23 (left) shows that a value of d = 2.3 Å gives the optimum distance, at approximately 0.5 eV. As expected, the pyridyl anchor group binds favourably to under-coordinated gold atoms. For SMe d = 2.7 Å gives the optimum distance, at approximately 0.4 eV.

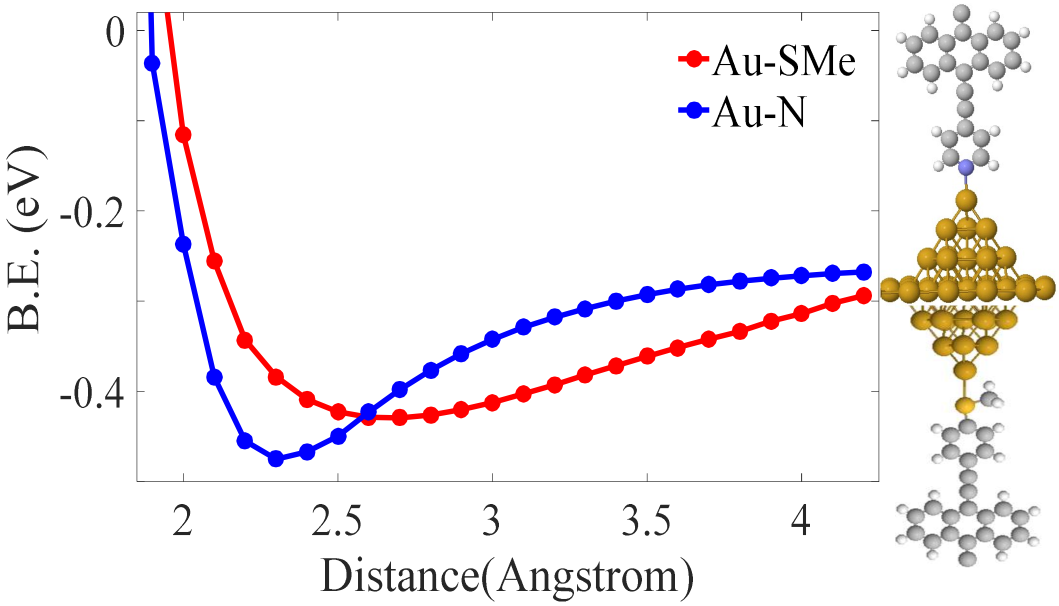

Figure S23: Example binding energy plot of **2** for two different anchors Au-N and Au-SMe (left), with its idealised adatom configuration at the Au lead interface (right, top Au-N and bottom Au-SMe). Key: C = grey, H = white, N = blue, S = light yellow, Au = dark yellow.

3.5 Optimised DFT structures of compounds within junctions

Using the optimised structures and geometries for the compounds obtained as described in section 2.1 (above), we again employed the SIESTA code to calculate self-consistent optimised geometries, ground state Hamiltonians and overlap matrix elements for each metal-molecule-metal junction. Leads were modelled as 625 atom slabs, terminated with 11-atom Au(111) tips. The optimised

structures were then used to compute the transmission curve for each compound. The DFT optimised geometries are shown here, in Figures S24-27. Note: there is a tilt angle range for each compound, which presents in section 2.5.

Key: C = grey, H = white, N = blue, S = light yellow, Au = dark yellow.

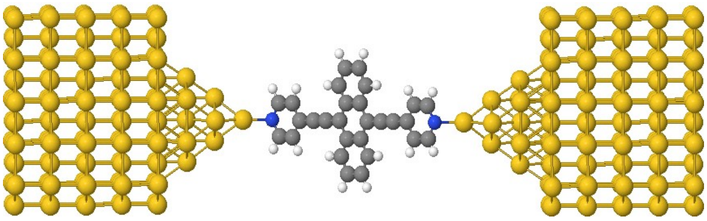

Figure S24: Optimised structure of **1**.

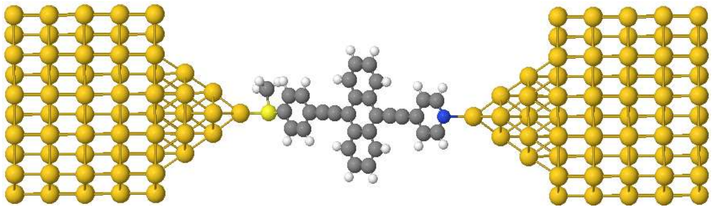

Figure S25: Optimised structure of **2**.

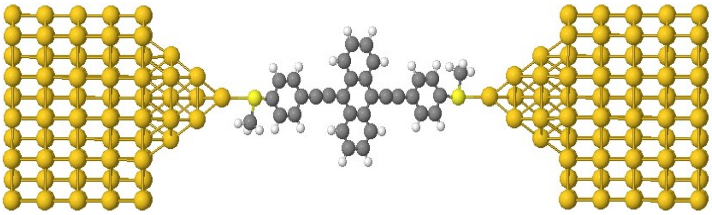

Figure S26: Optimised structure of **3**.

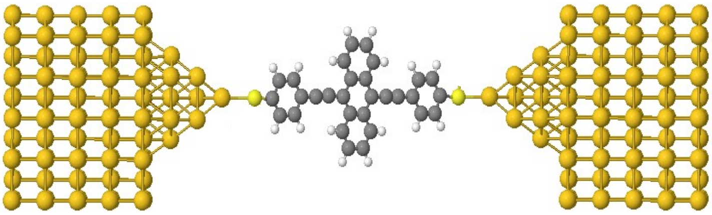

Figure S27: Optimised structure of **4**.

3.6 The tilt angle (θ)

In this section, we determine the tilt angle θ of each compound on a gold substrate, which corresponds to the experimentally measured most-probable break-off distance. Table S3 shows each compound for a range of tilt angles. Break-off distance values suggest that compound-**1** tilt with angle θ ranging from 40° to 60°, compound-**2** 41° to 53°, compound-**3** 51°-59° and compound-**4** 29°-33°

Table S3: Experimental break-off distance and equivalent tilt angle (θ)

Compound	Experimental film thickness (nm)	Experimental film roughness (nm)	Equivalent experimental tilt angle (θ)	Equivalent theoretical tilt angle (θ)
**1**	1.12	0.43	40°-60°	40°-60°
**2**	1.19	0.09	41°-53°	41°-53°
**3**	1.25	0.17	51 °-59°	51 °-59°
**4**	1.12	0.15	29°-33°	29°-33°

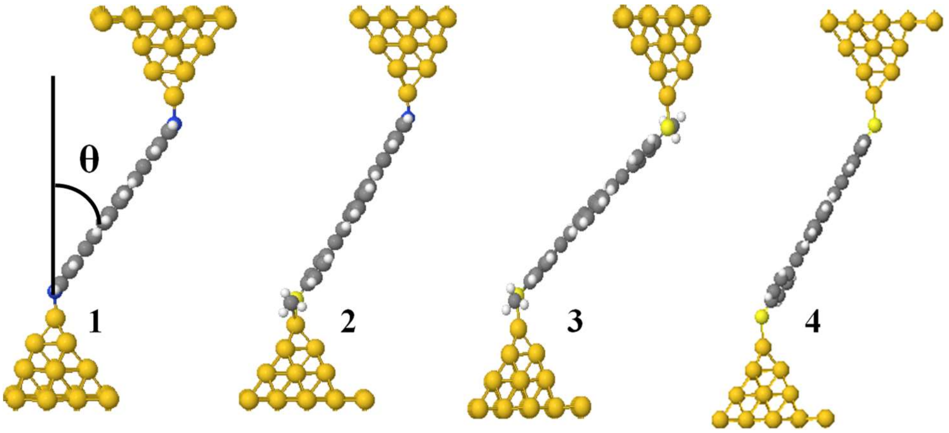

Figure S28: Optimised structures of **1-4**. Tilt angle (side-view)

3.7 HOMO-LUMO gaps

The calculated and optically measured HOMO-LUMO gaps are listed in Table S4. Theoretical gaps were calculated for isolated molecules and when the compounds are placed in the junctions, the gap between their HOMO and LUMO transmission resonances are quoted. As shown by the third and fourth columns in Table S4, isolated gaps for compounds **1**, **2**, **3** and **4** are larger than the gaps between the transmission resonances. This is because the latter are shifted by the real part of the self-energy of the contact to the leads, reflecting the fact that the system is more open when contacted to electrodes. In general, theoretical gaps are smaller than the measured gaps, which is consistent with the fact that DFT is known to underestimate its value.[15-16]

Table S4: Experimental and theoretical HOMO–LUMO gaps in eV.

Compound	Eg, (Exp.) a	Eg, DFT (Iso.) b	Eg, DFT (Au-M-Au)
1	2.66	1.64	1.50
2	2.61	1.60	1.45
3	2.57	1.50	1.40
4	2.66	1.51	1.20

[a] Experimental data: $E_g = 1241.5/\lambda_{ABS.}$ [b] Theoretical HOMO–LUMO gaps for the isolated molecules. [c] Theoretical gaps between HOMO–LUMO transmission resonances in Au|molecule|Au structures.

3.8 Transport calculations

The transmission coefficients *T(E),* obtained from using the Gollum transport code, were calculated for compounds **1-4** based on the tilt angle ranges in Table S3 (different curves of the same colour correspond to different title angles and the yellow line is the average). The LUMO resonance is predicted to be pinned near the Fermi Level of the electrodes for the four molecules, however, we choose Fermi Level to be in the vicinity of mid gap at ±0.5 eV (black-dashed line), as shown in Figure S29. Similarly, the conductance calculated for the same compounds as shown in Figure S29, yellow-lines indicate the average of each compound.

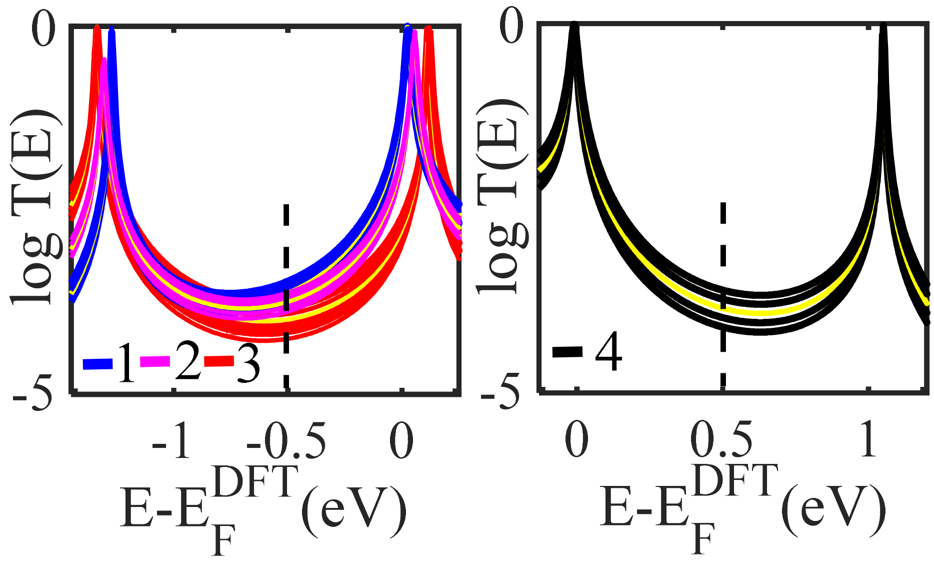

Figure S29: Transmission coefficients *T(E)* of molecules **1-4** against electron energy E, compound **1** (blue-line), compound **2** (pink-line), compound **3** (red-line), and compound **4** (black-lines). For each molecule, results are presented for 4 tilt angles in ranges shown in Table S3 Different curves of the same colour correspond to different title angles and the yellow line is the average of the four curves.

3.9 Seebeck coefficient

After computing the transmission coefficients of the four molecules, a study of their Seebeck coefficients S was made.

To calculate the Seebeck coefficient of these molecular junctions, it is useful to introduce the non-normalised probability distribution $P(E)$ defined by

$$P(E) = -\mathcal{T}(E)\frac{df(E)}{dE} \quad \text{(S2)}$$

where $f(E)$ is the Fermi-Dirac function and $\mathcal{T}(E)$ are the transmission coefficients and whose moments L_i are denoted as follows

$$L_i = \int dEP(E)(E - E_F)^i \quad \text{(S3)}$$

where E_F is the Fermi energy. The thermopower, S, is then given by

$$S(T) = -\frac{1}{|e|T}\frac{L_1}{L_0} \quad \text{(S4)}$$

where e is the electronic charge.

Supplementary Figure S30 shows the Seebeck coefficients S evaluated at room temperature for different energy range $E_F - E_F^{DFT}$.

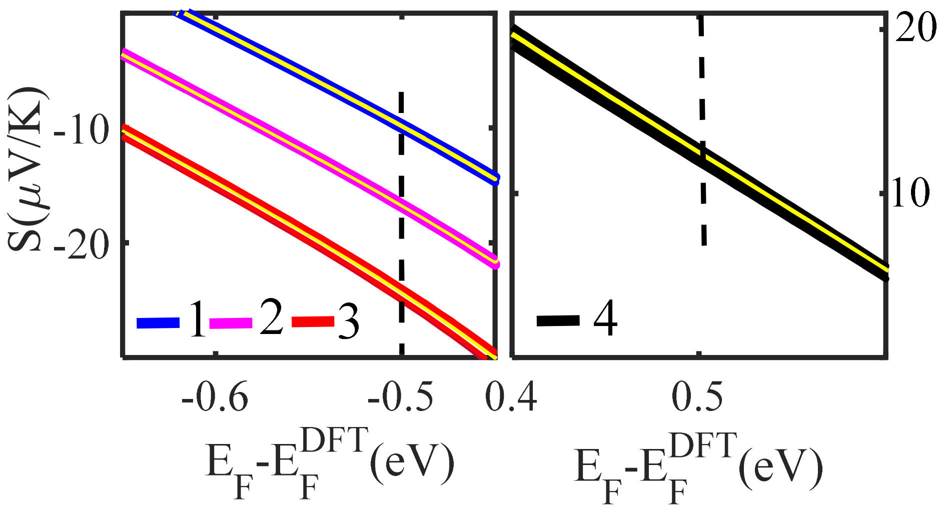

Figure S30: Seebeck coefficient S as a function of Fermi energy E_F for molecules 1 to 4 Results are shown for the tilt angles in the ranges shown in table S2 and the yellow line is the average.

3.10 Transport calculations for 1 and 2 (2Py and PySMe) at different anchor-electrode distances.

Figures S29 shows results at the optimum distances (d =0. 23 nm pyridyl and for SMe d =0. 27 nm, for **1**, **2**, **3** and **4**), between each anchor group and the electrodes. However, for **1** and **2** (2Py and PySMe) based SAMs, the theoretical conductances computed using the optimum distance (of 0.23 nm) between the anchor groups and electrodes are significantly higher than the measured values. This occurs, because as shown in Figures S16 and S17, the film quality of these SAMs is poorer than that of **3** and **4** (2SMe-, 2SAc-)-based SAMs. Consequently, the actual anchor-electrode distances in these SAMs is greater than the optimum value and measured to be of the order of 0.50 nm. Hence, for **1** and **2,** we show results as d varies from the optimum distance up to 0.50 nm. Figure S31 shows how the conductance decreases with increasing d, whereas the Seebeck coefficient is barely changed.

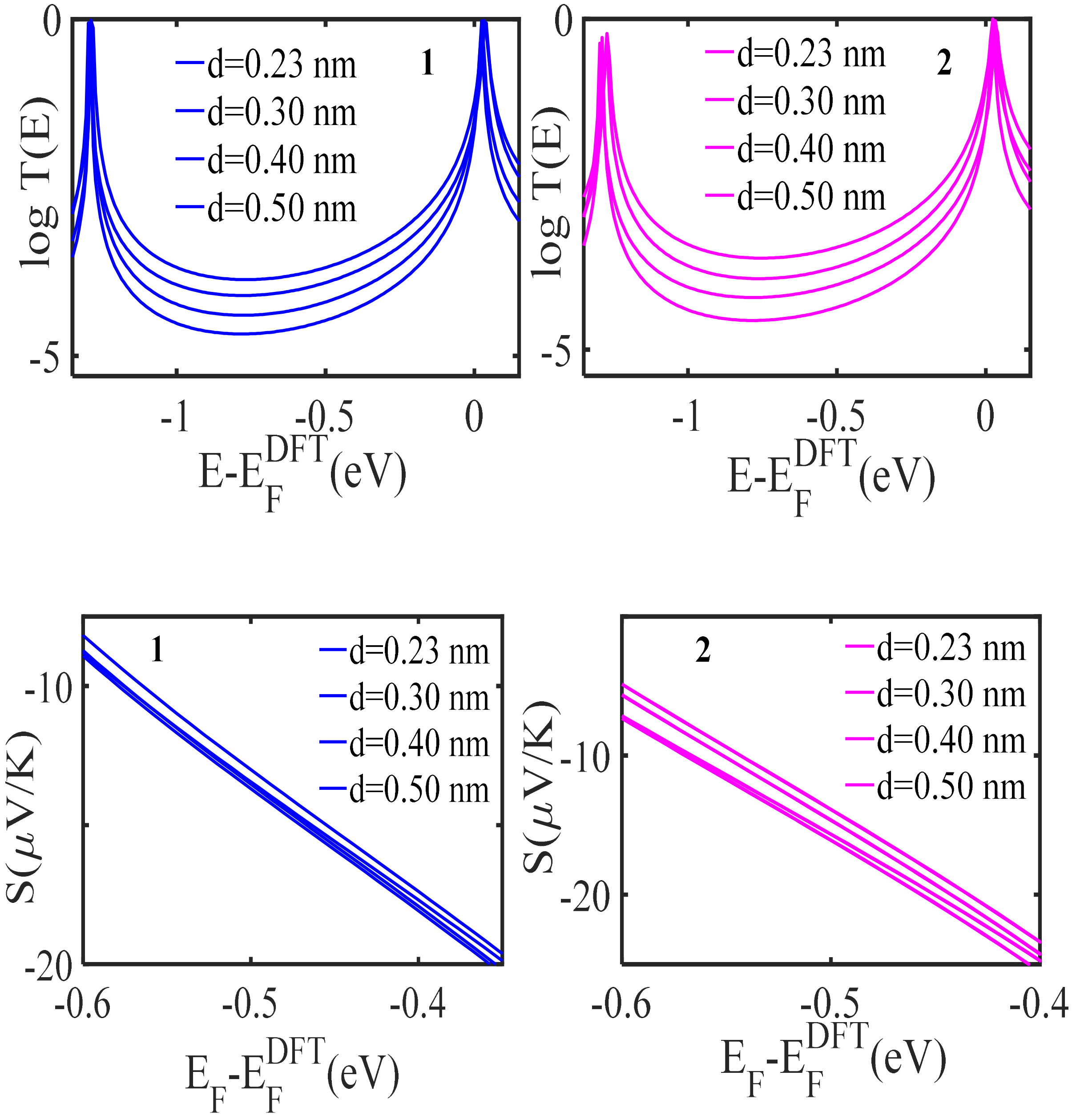

Figure S31: Zero bias transmission coefficient *T(E)* of molecules **1-2** against electron energy E, for different electrode-anchor distance d (**Top panel**). Seebeck coefficient **S** of the same molecules **1-2** (**middle panel**). Room-temperature Seebeck coefficients of **1**, **2, 3** and **4** evaluated for a range of the tilt angles (see Table S2), (**Lower panel**)

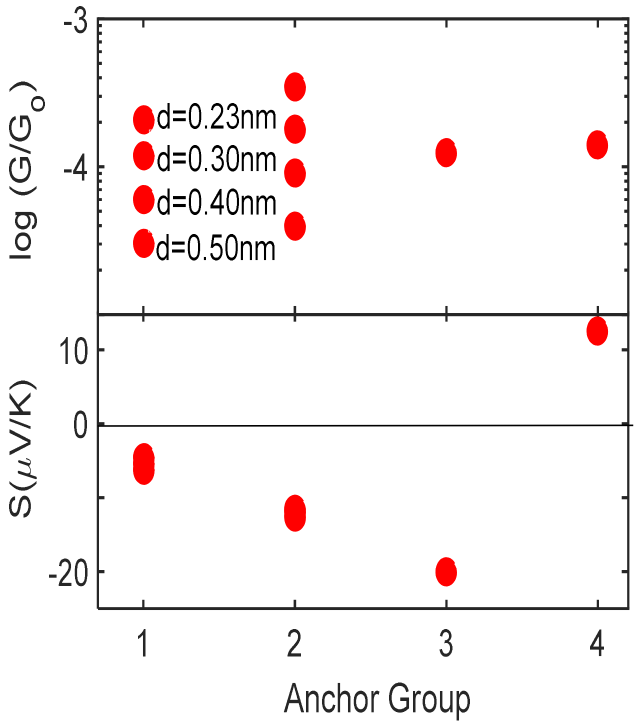

Figure S32: Electrical and thermoelectric properties of **1-4**. For **3** and **4** the optimum distance is 2.3 Å. For comparison, results are shown as this increases up to 5.0 Å.

4. References

1 S. H. Chanteau and J. M. Tour, Tetrahedron Lett., 2001, 42, 3057–3060.

2 W. Haiss, C. Wang, I. Grace, A. S. Batsanov, D. J. Schiffrin, S. J. Higgins, M. R. Bryce, C. J. Lambert and R. J. Nichols, Nat. Mater., 2006, 5, 995–1002.

3 L. E. Wilson, C. Hassenrück, R. F. Winter, A. J. P. White, T. Albrecht and N. J. Long, Eur. J. Inorg. Chem., 2017, 2017, 496–504.

4 V. Kaliginedi, P. Moreno-García, H. Valkenier, W. Hong, V. M. García-Suárez, P. Buiter, J. L. H. Otten, J. C. Hummelen, C. J. Lambert and T. Wandlowski, J. Am. Chem. Soc., 2012, 134, 5262–5275.

5 P. Nguyen, S. Todd, D. Van den Biggelaar, N. J. Taylor, T. B. Marder, F. Wittmann and R. H. Friend, Synlett, 1994, 4, 294–301.

6 H. S. Quah, V. Nalla, K. Zheng, C. A. Lee, X. Liu and J. J. Vittal, Chem. Mater., 2017, 29, 7424–7430.

7. Soler, J. M.; Artacho, E.; Gale, J. D.; García, A.; Junquera, J.; Ordejón, P.; Sánchez-Portal, P., The SIESTA method for ab initio order- N materials simulation. *J. Phys.: Condens. Matter* 2002, 14 (11), 2745.

8. Artacho, E.; Anglada, E.; Diéguez, O.; Gale, J. D.; García, A.; Junquera, J.; Martin, R. M.; Ordejón, P.; Pruneda, J. M.; Sánchez-Portal, D.; Soler, J. M., The SIESTA method; developments and applicability. *J. Phys.: Condens. Matter* 2008, 20 (6), 064208.

9. Herrer, I. L.; Ismael, A. K.; Milán, D. C.; Vezzoli, A.; Martín, S.; González-Orive, A.; Grace, I.; Lambert, C.; Serrano, J. L.; Nichols, R. J.; Cea, P., Unconventional Single-Molecule Conductance Behavior for a New Heterocyclic Anchoring Group: Pyrazolyl. *The Journal of Physical Chemistry Letters* 2018, 9 (18), 5364-5372.

10. Ismael, A. K.; Wang, K.; Vezzoli, A.; Al-Khaykanee, M. K.; Gallagher, H. E.; Grace, I. M.; Lambert, C. J.; Xu, B.; Nichols, R. J.; Higgins, S. J., Side-Group-Mediated Mechanical Conductance Switching in Molecular Junctions. *Angew. Chem. Int. Ed.* 2017, 56 (48), 15378-15382.

11. Andrei, M.; Ali K. Ismael, Ross J. Davidson, David C. Milan, Richard J. Nichols, Simon J. Higgins, Colin J. Lambert, Yu-Ting Hsu, Dmitry S. Yufit, and Andrew Beeby. "Conductance Behavior of Tetraphenyl-Aza-BODIPYs." *The Journal of Physical Chemistry C* (2020).

12. Yoshizawa, K.; Tada, T.; Staykov, A., Orbital views of the electron transport in molecular devices. *Journal of the American Chemical Society* 2008, 130 (29), 9406-9413.

13. Li, X.; Staykov, A.; Yoshizawa, K., Orbital Views of the Electron Transport through Polycyclic Aromatic Hydrocarbons with Different Molecular Sizes and Edge Type Structures. *The Journal of Physical Chemistry C* 2010, 114 (21), 9997-10003.

14. Tsuji, Y.; Staykov, A.; Yoshizawa, K., Orbital views of molecular conductance perturbed by anchor units. *Journal of the American Chemical Society* 2011, 133 (15), 5955-5965.

15. Hung, Y.-C.; Jiang, J.-C.; Chao, C.-Y.; Su, W.-F.; Lin, S.-T., Theoretical Study on the Correlation between Band Gap, Bandwidth, and Oscillator Strength in Fluorene-Based Donor–Acceptor Conjugated Copolymers. *The Journal of Physical Chemistry B* 2009, 113 (24), 8268-8277.

16. Lof, R. W.; van Veenendaal, M. A.; Jonkman, H. T.; Sawatzky, G. A., Band gap, excitons and Coulomb interactions of solid C60. *J. Electron. Spectrosc. Relat. Phenom.* 1995, 72, 83-87.
